# Development of a SNP Amplicon Panel for Invasive Deer Faecal Pellets and the Effects of Environmental Exposure on Genotyping Success

**DOI:** 10.1002/ece3.74388

**Published:** 2026-09-21

**Authors:** Tamandra H. D'Ombrain, Katherine A. Harrisson, Carlo Pacioni, Erin Hill, Nicholas P. Murphy

**Affiliations:** ^1^ Department of Ecological, Plant and Animal Sciences La Trobe University Bundoora Victoria Australia; ^2^ Department of Energy, Environment and Climate Action Arthur Rylah Institute for Environmental Research Heidelberg Victoria Australia; ^3^ Health and Biosecurity CSIRO Canberra Australian Capital Territory Australia

**Keywords:** faecal degradation, GT‐seq, invasive species, non‐invasive monitoring, sambar deer (*Cervus/Rusa unicolor*), scats

## Abstract

Non‐invasive genetic sampling techniques hold great promise for wildlife monitoring but pose considerable challenges for obtaining genetic data. For faecal samples, mixed DNA from many organisms, low concentration, reaction inhibitors, and DNA degradation over time often hinder success. We aimed to develop a high‐throughput, cost‐effective faecal genotyping technique for monitoring invasive sambar deer ( *Cervus unicolor* ). We developed a multiplexed amplicon panel targeting 371 single nucleotide polymorphism (SNP) markers previously genotyped using restriction‐site associated DNA sequencing, and three sex markers. To evaluate panel performance in faecal samples, we undertook a degradation trial assessing the effects of time (0, 2, 4, 7, and 14 days) on genotyping success under three conditions (outdoors on exposed ground, outdoors protected from rain and UV, and indoors). Fresh faecal samples performed equally to tissue, but environmental exposure rapidly degraded DNA. On average, exposed pellets were viable up to 4 days, with ~200 SNPs successfully genotyped, while protected samples were still viable after 14 days. In the field, we recommend sampling when there has been no rain for several days. We demonstrate that our method reliably ascertains individuals, sex, and species, with sufficient concordance among sample types and genotyping methods to support integration of faecal‐derived genotypes with existing datasets. Using existing reduced‐representation tissue sample data, we further demonstrate that the 200 SNPs reliably genotyped can resolve population structure and first‐order kinship, aiding informed management. More broadly, the insights for cost‐effective faecal DNA genotyping with optimised sample collection will benefit monitoring of both native wildlife and invasive species.

## Graphical abstract

We developed a SNP panel for non‐invasive genetic monitoring of introduced sambar deer which enables the integration of faecal‐derived genotypes with existing data. Our degradation study found that exposed faecal samples were viable up to 4 days, with ~200 SNPs successfully genotyped, while protected samples were still viable after 14 days. Our panel provides sufficient resolution for ascertaining species, sex, individuals, high‐level population structure and first‐order kinship in this species, aiding informed management.

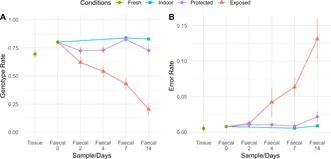

## 1 Introduction

The conservation of biodiversity and ecosystems is a task of increasing importance and complexity, where efficient use of limited resources is crucial (Rands et al. 2010; Wilson et al. 2007). Effective wildlife management relies on accurate monitoring data, with genetic techniques becoming increasingly powerful and cost‐effective for improving monitoring (Piertney 2016; Primmer 2009). Genetic applications include species detection, population demography and movement, genetic diversity, taxonomy, disease, diet and forensics (reviewed by Hohenlohe et al. 2021; Steiner et al. 2013). Sample types for acquiring DNA range from invasive sampling of blood or tissue (Sherwin 1991), less invasive methods such as hair or swabs (Ford et al. 2016; Peralta et al. 2019), to completely non‐invasive samples including faeces (Lucchini et al. 2002), shed skin (Brekke et al. 2023), or environmental DNA (Bohmann et al. 2014; Taberlet et al. 2012). Although posing some sequencing challenges, non‐invasive methods offer improved sampling of species that are otherwise difficult or costly, and enable ethical sampling of living specimens (Carroll et al. 2018; Waits and Paetkau 2005).

Faecal samples are appealing for non‐invasive genetic studies due to their availability in the environment and relative ease of attributing to species. Faecal samples have previously been unsuitable for many genomic applications due to low quantity DNA of mixed origin (including diet and microorganisms), presence of reaction inhibitors, and DNA degradation from environmental exposure (Carroll et al. 2018; Taberlet et al. 1999). However, with next generation sequencing advances (Piertney 2016), improved collection protocols to prioritise target species DNA (Ramón‐Laca et al. 2015), and availability of genomic resources for species‐specific approaches (Genome 10 K Community of Scientists 2009), opportunities to utilise faecal samples continue to improve.

Single nucleotide polymorphism (SNP) markers have overtaken microsatellites as the preferred marker for population genetics, due to their analytical power, ease of amplification, consistency between laboratories, and low genotyping error (Helyar et al. 2011; Morin et al. 2004). Reduced‐representation sequencing approaches (e.g., GBS, RADseq, DArTseq) can generate thousands of SNPs without requiring a reference genome, but may be prohibitively expensive for conservation programs, and generally require good quality, uncontaminated DNA. Targeted SNP panels such as ‘Genotyping‐in‐Thousands by sequencing’ (GT‐seq; Campbell et al. 2015) provide a cost‐effective alternative using custom amplicon sequencing of hundreds of loci via multiplexed polymerase chain reaction (PCR). Species‐specific primers and relatively short fragments allow GT‐seq to be applied to low quality/quantity DNA containing non‐target material, making it suitable for minimally invasive and/or degraded samples. Variations of GT‐seq have been applied successfully to rattlesnake cloacal swabs (Schmidt et al. 2020), archival fish scales (Setzke et al. 2021), porpoise carcasses (Morin et al. 2021), cooked conch fritters (Natesh et al. 2019), and faecal samples from multiple mammalian species, including coyotes (Eriksson et al. 2020), polar bears (Hayward et al. 2022), tigers (Natesh et al. 2019) and deer (Burgess et al. 2022). However, using faecal samples for large‐scale genetic monitoring requires understanding DNA viability under different environmental conditions.

Despite the increasing use of faecal samples with GT‐seq panels (Arpin et al. 2024; Burgess et al. 2022; Eriksson et al. 2020; Hayward et al. 2022), knowledge gaps remain around degradation impacts. Degradation of DNA generally refers to a reduction in fragment length; however, the DNA quantity recovered may also decrease with environmental exposure (Cowen et al. 2022; Wedrowicz et al. 2013). Studies using microsatellites have suggested a negative impact of time (Agetsuma‐Yanagihara et al. 2017; Carpenter and Dziminski 2017; Panasci et al. 2011; Piggott 2004; Woodruff et al. 2015), rainfall (Agetsuma‐Yanagihara et al. 2017; Brinkman et al. 2010; Wedrowicz et al. 2013) and solar exposure (radiant solar energy in MJ/m^2^; Cowen et al. 2022) on faecal genotyping success. Furthermore, visual appearance can be an unreliable indicator of sample quality (Agetsuma‐Yanagihara et al. 2017; Piggott 2004), and DNA preservation and extraction methods also influence genotyping success (Panasci et al. 2011; Piggott and Taylor 2003). However, DNA requirements likely vary among sequencing technologies, with approaches that use shorter fragment lengths being more robust to degraded DNA (Hughes‐Stamm et al. 2011). Relatively few SNP‐based faecal degradation studies have been performed, with inconsistent findings reported (Levengood et al. 2025; Scholz et al. 2024; Schultz et al. 2018). These studies used varying genotyping methods, namely restriction‐site associated reduced‐representation sequencing (DArTseq; Schultz et al. 2018), a Fluidigm microarray platform (Scholz et al. 2024), and a targeted bait‐capture method (DArTcap; Levengood et al. 2025). Degradation effects are yet to be studied for GT‐seq faecal genotyping.

Introduced deer in southeastern Australia offer an opportunity to explore the feasibility of non‐invasive genetic wildlife monitoring. Arising from acclimatisation society introductions, translocations, and farm releases beginning in the 19th century, deer are now widespread in Australia (Moriarty 2004) and effective management of the six established species is essential to minimising their impacts (reviewed by Davis et al. 2016). However, management can be hindered by their elusive nature, often necessitating time consuming or expensive monitoring techniques (Amos et al. 2014; Hampton et al. 2019). Genetic studies are increasingly recognised as critical to understand dispersal and management units of invasive species (Le Roux and Wieczorek 2009), and studies of deer in Australia have typically utilised population control and recreational hunting for genetic samples (Hill, Murphy, Li‐Williams, et al. 2023; Hill, Murphy, Linacre, et al. 2023; Li‐Williams et al. 2023; Rollins et al. 2023). Thus, sampling is largely opportunist and completely shaped by where such activities are occurring. Furthermore, studies limited to deceased deer preclude analyses which require repeated sampling of individuals, including mark‐recapture population estimates (Li et al. 2020), movement tracking (Lucchini et al. 2002), or comparisons before and after population control (Piggott et al. 2008). Faecal pellets are a readily collectable resource enabling targeted, non‐invasive sampling of living individuals—if deer pellets can be reliably genotyped.

Sambar deer ( *Cervus unicolor* ) are the most abundant deer species in Victoria, Australia, with a 2023 statewide estimate of 123,061 individuals inhabiting public‐tenured land (Cally and Ramsey 2023). Large‐scale sambar deer genetic monitoring has been established using DArTseq (a double digest restriction‐site associated DNA sequencing method; Kilian et al. 2012), incorporating a decade of opportunistic tissue sampling (Hill, Murphy, Li‐Williams, et al. 2023; Hill et al. unpublished data). Faecal samples have also been used in previous studies to successfully obtain microsatellites (11 loci; Davies et al. 2020) and SNPs (34 amplicons; White et al. 2025). However, faecal samples could be better utilised if markers were designed using known Australian sambar variants, amplified in a streamlined multiplex PCR, had the power to resolve population structure and kinship in addition to sex, species and individual identification, and could be combined for analysis with the wealth of existing DArTseq SNP data. We selected GT‐seq for this purpose over capture‐based methods due to relatively low cost and accessible laboratory processing. However, in selecting target SNPs, a balance is required between panel size, sample retention (given faecal degradation), and species‐specific genetic constraints including hybridisation with rusa deer ( *Cervus timorensis* ; Hill, Murphy, Li‐Williams, et al. 2023), related individuals (Hill, Murphy, Li‐Williams, et al. 2023), and low genetic diversity resulting from the introduction of relatively few individuals (Rollins et al. 2023). A DArTseq‐compatible, high‐throughput method for genotyping faecal samples would transform sambar deer monitoring.

Here, we developed a cost‐effective GT‐seq based method for genotyping sambar deer faecal samples to support monitoring and management of invasive populations. To understand the effects of environmental exposure on faecal genotyping success, we performed a degradation study including both field and lab conditions. Specifically, this study aimed to: (1) design a SNP panel for sambar deer with capability for determining individuals, species, sex, population structure and kinship; (2) evaluate faecal pellet DNA degradation under different levels of environmental exposure and the effects on genotyping and individual identification; (3) compare genotyping success and concordance among sample types (faecal and tissue) and sequencing methods (amplicon and DArTseq) to determine suitability for co‐analysis with existing DArTseq SNP data; and (4) using DArTseq data with the subset of SNPs reliably genotyped by the panel, compare the population structure and kinship inferences with those of a larger dataset.

## 2 Materials and Methods

### 2.1 Panel Design and Development

For compatibility with existing data, we designed amplicons based on multiple DArTseq SNP datasets generated from 739 tissue and blood samples of sambar, rusa and fallow deer in Australia, for which sampling information and population genetic analyses are described in the associated publications (Hill, Murphy, Li‐Williams, et al. 2023; Li‐Williams et al. 2023; White et al. 2026). These DArTseq data were used for development and evaluation of our SNP panel. Panel design and development was an iterative process involving multiple Illumina MiSeq sequencing rounds, during which data were processed primarily in *R* (≥ v4.2; R Core Team 2022) using *dartR*/*dartRverse* packages (Gruber et al. 2018; Mijangos et al. 2022) and *tidyverse* core packages (Wickham et al. 2019) unless otherwise stated.

Prior to amplicon development, we first delimited species and hybrids in the DArTseq data using a *Structure* v2.3.4 (Pritchard et al. 2000) analysis (Material S1). Following species and hybrid identification, we performed exploratory simulations to evaluate the number of SNPs required for individual identification in introduced sambar deer (Material S2). Simulations incorporating varying minor allele frequency, number of SNPs and error rate (Material S2) suggested a panel of at least 200 highly variable SNPs would enable robust individual assignment. In primer design, we targeted approximately double this number to buffer against the loss of loci during optimisation and filtering (May et al. 2020).

To select SNPs for designing primers, we first retained loci with 100% reproducibility among DArT technical replicates and 98% locus call rate from 528 samples of sambar, rusa and hybrids. Data were then subset to ‘pure’ sambar individuals (Material S1) and loci with minor allele frequency (MAF) > 0.2, with the addition of some DArTseq loci that were putatively fixed between sambar and rusa species. These steps ensured the selected loci were highly variable in sambar, and had high‐quality data in rusa and hybrid samples for co‐analysis with faecal genotypes. A red deer ( *Cervus elaphus* ) reference genome was used for primer design, the closest chromosome‐level assembly available. The 69 bp sambar DArTseq sequences were aligned to the autosomal chromosomes of the mCerEla1.1 genome (assembly GCA_910594005.1) and extended by 150 bp either side using *BWA* v0.7.17 (Li and Durbin 2009), *SAMtools* v1.15.1 (Li et al. 2009) and *BEDTools* v2.30.0 (Quinlan and Hall 2010) under strict parameters (minimum alignment score 60, mapping quality 30 and query length 65, remaining parameters default).

Primers were designed for the extended sequences using *FastPCR Web Tools* v3.6.17 (Kalendar et al. 2017, 2011) to produce amplicons containing the 69 bp sequence. Primers were searched against the mCerEla1.1 genome using *BLAST* (Altschul et al. 1990), and any with multiple hits were redesigned or discarded. After sequencing the first panel iteration, a second round of primer design was performed, with MAF reduced to > 0.1 to increase the number of candidate loci. In silico primer dimer analysis was conducted in *FastPCR*, and primers with strong interactions were removed. As sex information is important for deer management, previously published length‐based sex markers were included in initial panel testing (Table S1). However, the X and Y amplification ratios were deemed unsuitable, and lengths varied between species. For more accurate and streamlined sex genotyping, we subsequently designed primer sets for three new sex markers using *Geneious Prime* v2022 by finding conserved regions between X and Y chromosomes in deer and other Artiodactyls, where primers matched exactly to both chromosomes but amplified fragments with a putatively fixed SNP difference (Tables S2–S9).

Initial sequencing runs trialled multiple subsets of loci to evaluate amplification success and multiplex size impacts. Primers were initially tested using premixed pools (Integrated DNA Technologies Opools) and subsequently ordered individually (from Macrogen) to allow for adjustment of combinations. Testing was conducted using tissue samples (*n* = 38 individuals) from sambar, rusa, sambar‐rusa hybrids, fallow and red deer, with all samples except red deer previously sequenced using DArTseq (Figure S1).

### 2.2 Degradation Study

Sambar deer colon sections containing faecal material were collected by Parks Victoria during 2023 control operations in northeast Victoria (Mount Granya State Park and Mount Lawson State Park) and stored at approximately −20°C without preservative until experiment set up (4 months). Although introducing some potential differences from naturally voided pellets (e.g., microbial composition and storage effects), using colon faeces allowed the inclusion of paired tissue for estimating faecal error, and a uniform starting time for exposure in our degradation study. For each colon, a tissue sample was collected, and faecal pellets were carefully removed, avoiding visible blood to prevent inflating DNA presence. Pellets were distributed among three experimental conditions—exposed, protected, and indoor. Exposed and protected pellets were placed outdoors on grass in the La Trobe University Agricultural Reserve, with the protected group placed underneath a 99% UV protective polycarbonate sheet (SunTuf Sunlite 8 Twinwall Clear) held 20 cm above the ground to allow light, air flow and humidity fluctuation but limit rain and UV exposure. Indoor samples were kept in large petri dishes with lids removed, in a PCR‐free laboratory at room temperature. We first conducted a pilot study (*n* = 3 individuals) to explore the appropriate sampling intervals, with faecal DNA samples collected after 0, 7, and 14 days. We subsequently performed the full experiment (*n* = 20) with samples collected after 0, 2, 4, 7 and 14 days in October 2023. Weather observations for the study period were retrieved from the Bureau of Meteorology Viewbank (086068) weather station (~5 km from the site). Minimum daily temperature ranged from 4.2°C–17.2°C with a mean of 9.5°C, while maximum daily temperature ranged from 15.1°C–28.8°C with a mean of 20.2°C. Rain was observed on 6 days, with 35.6 mm total rainfall. Days 4 and 7 both recorded 13 mm rainfall in the 24 h prior to 9 am. Mean relative humidity at 9 am was 82.7%, with 1 day recording 100%. Temperature and rainfall were typical for October, while relative humidity was slightly above average (Australian Bureau of Meteorology 2026).

Faecal pellets were swabbed for DNA based on the methods of Ramón‐Laca et al. (2015) and Davies et al. (2020). For each sample, a single faecal pellet was picked up using a toothpick, a sterile cotton‐tipped wooden swab was dipped into a microcentrifuge tube prefilled with 500 μL Longmire buffer (Longmire et al. 1997), and the entire surface was swabbed until a colour change was observed all over the swab. For samples which had dried out (particularly indoor), this process took 10 s or more of vigorous swabbing, while for fresh samples or those collected after rain, pellets were fragile and required gentle swabbing to prevent disintegration or excess faecal material collection. This swabbing variation may influence DNA yield but was necessitated by pellet variation. Swab heads were detached with scissors, returned to their tube of Longmire buffer, and stored at 4°C for 4–12 days before DNA extraction. All time and treatment combinations for 20 individuals were collected (*n* = 280), but not all were deemed necessary based on pilot study results (Figure S2). Samples from 16 individuals were processed (*n* = 160, Table 1) to manage sequencing depth without biassing conclusions. As indoor samples showed limited DNA degradation in the pilot study, we excluded Days 2 and 4 and processed 8/16 samples for Day 7. As exposed samples demonstrated substantial degradation, and samples comprising mostly primer dimer reduce overall sequencing run quality, we processed 8/16 samples for Day 14. To explore how degradation influenced our ability to identify individuals, we included seven replicate samples (i.e., one DNA extract used in two PCR reactions) chosen across the time series. To examine the effects of reduced DNA input (as opposed to degradation) we diluted six samples from tissue (*n* = 4) and Day 0 faecal (*n* = 2) to a starting DNA concentration of 0.5 ng/μL (Table 1).

**TABLE 1:** Samples (replicates, dilutions) sequenced for each time/condition combination.

	Tissue	Faecal Day 0	Faecal Day 2	Faecal Day 4	Faecal Day 7	Faecal Day 14
Fresh	16 (2,4)	16 (0,2)				
Indoor			—	—	8 (0,0)	16 (1,0)
Protected			16 (0,0)	16 (1,0)	16 (0,0)	16 (0,0)
Exposed			16 (1,0)	16 (1,0)	16 (1,0)	8 (0,0)

Faecal swab and colon tissue DNA extractions both used Qiagen reagents and EZ‐10 spin columns (Bio Basic SD5005), with differences in lysis procedure, incubation and elution volume. Swab extractions were performed in a separate PCR‐free laboratory, based on the Qiagen QIAamp DNA Mini protocol ‘DNA Purification from Buccal Swabs’, and published methods (Davies et al. 2020; Ramón‐Laca et al. 2015). Proteinase K (20 μL) and Buffer AL (500 μL) were added directly to tubes containing swab heads in Longmire buffer (Longmire et al. 1997). After 56°C lysis for 15 min, 500 μL ethanol was added, and 1200 μL of the resultant solution was processed through spin columns followed by standard wash steps. The DNA was eluted in 100 μL of molecular grade water after 1 h incubation. Tissue extractions followed the Qiagen DNeasy Blood & Tissue ‘Purification of Total DNA from Animal Tissues’ protocol, with DNA eluted in 200 μL after a 20‐min incubation. Extracted DNA from faecal and tissue samples was stored at −20°C until library preparation (< 1 month).

### 2.3 Library Preparation and Sequencing

Library preparation was based on the GT‐seq method (Campbell et al. 2015) with some modifications. Adjustments were made to the protocol during panel development, and the final library preparation and sequencing (the degradation study) used the following procedure.

Tissue and swab DNA extracts were quantified with a NanoDrop Lite Spectrophotometer (Thermo Fisher) and normalised to 10 ng/μL and 5 ng/μL, respectively (except 14 lower concentration swab extracts and the dilution samples). NanoDrop quantification was selected over fluorometric methods at this stage for cost‐effectiveness. Multiplex PCR of the amplicon panel contained 5 μL of Qiagen Multiplex PCR Master Mix, 1 μL of pooled primers (at 250 nM per primer; Table S10), and either 4 μL of DNA (for samples ≤ 5 ng/μL) or 2 μL H_2_O and 2 μL of DNA (for 10 ng/μL samples), bringing the reaction total to 10 μL. The PCR1 conditions were 95°C—15 min; 20 cycles of 94°C—30 s, 60°C—90 s, 72°C—90 s; 72°C—10 min; 4°C hold. The relatively low 20 PCR cycles were selected to reduce PCR error and primer dimers. Resultant PCR products were cleaned individually using solid‐phase reversible immobilisation (SPRI) magnetic bead mix to reduce primer dimer and eluted in 20 μL of H_2_O (two‐fold dilution). Indexing PCR was undertaken in 15 μL comprising 7.5 μL GoTaq Green Master Mix, 3.5 μL H_2_O, 1 μL index primer pair (at 10 μM per primer) and 3 μL of cleaned PCR product. The PCR2 conditions were 95°C—3 min; 10 cycles of 95°C—30 s, 60°C—30 s, 72°C—30 s; 72°C—5 min; 4°C hold. Resultant PCR2 products were again cleaned individually with SPRI beads. The inclusion of bead cleans after both PCRs, a divergence from the GT‐seq protocol (Campbell et al. 2015) which included only a (pooled) clean after PCR2, was shown by Eriksson et al. (2020) to greatly improve the proportion of on‐target reads and reduce index hopping in faecal samples, compared to a single bead clean. Cleaned PCR2 products were quantified using Qubit dsDNA HS Assay Kit reagents with either a Qubit 2.0 Fluorometer (Invitrogen) or CLARIOstar plate reader (BMG LABTECH) and the nM concentration was calculated based on expected average amplicon size (noting this is less exact than direct library measurement). Samples were normalised to estimated 2 nM where possible (52 low concentration samples were used unadjusted) and pooled into four pools. Following an initial poor sequencing run due to off‐target sequences skewing final concentrations (noting this was due to the intentional sequencing of low quality, degraded samples), a final bead clean was performed to further reduce these fragments. The final library was prepared as per Illumina MiSeq protocol and sequenced as part of a MiSeq Reagent Kit v3 run with paired end reads (2 × 200 bp).

### 2.4 Genotyping and Genetic Analysis

Demultiplexed reads were trimmed and merged based on overlap using *PEAR* v0.9.11 (Zhang et al. 2014). Parameters ‘‐v 15 ‐m 290 ‐n 100 ‐t 100 ‐q 10’ were used, corresponding to a trimming quality score of 10, minimum read length of 100 after trimming, minimum read overlap of 15 and assembled length minimum of 100 and maximum of 290. Loci were then genotyped using the GTscore pipeline (McKinney et al. 2020). To produce the GTscore ‘probe’ sequences used for calling SNPs, we aligned reads to the expected PCR products (extracted from the mCerEla1.1 genome) in *Geneious Prime* and generated consensus sequences representing the variation within the reads and the DArTseq sequences (Table S11). Probes of 11 bp containing the target SNP (in the direction of the DArTseq data) were exported, with variable bases specified (Table S12). Probes for the three sex SNP markers were based on the expected fixed SNP differences (Table S12). After genotyping, the SNP data were converted to genlight format (Jombart 2008) for analysis. Scripts and packages used are available online (https://doi.org/10.26181/31302583).

Faecal degradation analyses were performed prior to genotype rate filtering to retain degraded samples in the dataset and accurately represent genotyping variation under different levels of exposure. Faecal sample error rate was calculated as the genetic distance between each faecal sample and its reference tissue sample (considered the ‘true’ genotype). Genetic distances (as a proportion of alleles) were calculated using the function ‘bitwise.dist’ from the *poppr* package (Kamvar et al. 2014), based on loci for which both samples were genotyped (i.e., missing data was excluded rather than considered a match). Tissue error rate was calculated from replicates (*n* = 2); other analyses of genotype rate, error rate and sex determination excluded replicates and dilutions. The effects of time and environmental conditions on genotyping and error rate were tested using Bayesian regression models in the *brms* package (Bürkner 2017) with default priors. A beta regression model was fitted with genotype rate as the response variable, while xbeta (a regression model with an underlying beta distribution which can handle 0 or 1 values) was fitted for error rate. Four independent chains were used to fit the models, which were considered converged if the Gelman‐Rubin statistic (Brooks and Gelman 1998) was 1. The ‘loo’ function was used to compare models and determine the best fit (Material S3). For the selected models, day and condition were fixed effects and the interaction term was included. To account for repeated sampling of pellets from the same individuals across time, day was included as a random slope and individual as a random intercept. The base (reference) model represented the indoor condition. Sex determination accuracy (compared to recorded sex) in the degradation study samples was assessed for each sex marker individually, and with a combined approach where sex was assigned only if at least 2/3 loci were genotyped and no genotypes conflicted.

To determine the appropriate filtering and genetic distance thresholds for individual identification, we explored various genotype rate or read depth filtering and plotted pairwise genetic distance, aiming to produce a clear separation between comparisons from the same individual and those of distinct individuals. Replicates and dilutions were retained here to inform within‐individual variation. The filtering steps subsequently selected for robust individual identification were to first remove samples with a genotype rate < 50% (187/374 loci), followed by loci with genotype rate < 80%. These filtering thresholds allowed for a standardised approach with high confidence in individual assignment for future panel applications. Samples excluded in filtering were deemed to have insufficient resolution for ascertaining individuals.

### 2.5 Panel Evaluation

To assess both genotype discordance and the power of a reduced marker set generated from the amplicon panel relative to DArTseq, we identified SNP loci reliably genotyped with our panel. We first excluded loci with > 50% disagreement between amplicon and DArTseq genotypes for the 25 sambar samples, due to presumed different genomic positions rather than sequencing error. We then retained the 200 polymorphic autosomal SNP loci with the highest genotyping success in fresh faecal samples and panel development samples (to include population variation). We used these 200 SNPs in samples which passed filtering to assess genotyping discordance within individuals across sample types (faecal and tissue) and genotyping methods (amplicon and DArTseq). Discordance was calculated as genetic distance (using ‘bitwise.dist’ without one sample considered ‘correct’ as in error rate calculations). Replicates and dilutions which passed filtering were included.

To understand how the amplicon panel SNPs reliably genotyped from faecal samples would perform for population structure and kinship analyses, we retrospectively analysed the DArTseq data used to develop the panel. We filtered the sambar dataset for reproducibility (threshold = 0.99), read depth (between 5 and 100), locus callrate (threshold = 0.95), individual callrate (threshold = 0.75), minor allele count (≥ 3), and secondaries (‘best’ method selected), with any monomorphic loci removed, resulting in 326 samples and 7412 SNPs, termed the ‘7412 SNP dataset’. We then subsampled the DArTseq data for the same 326 samples to retain only our 200 reliable SNPs, termed the ‘200 SNP dataset’, and compared results to the larger filtered dataset. To compare the relative resolving power, we ran *Structure* (Pritchard et al. 2000) for both the 200 and 7412 SNP DArTseq datasets, with 10 replicates per *K* from 1 to 10, 100,000 burnin and 500,000 MCMC iterations. The *Pophelper* R package (Francis 2017) was used to summarise the outputs, and the Δ*K* method (Evanno et al. 2005) and ln Pr(X|*K*) method (Pritchard et al. 2000) for determining the best *K* were compared. Population structure was previously explored for these DArTseq data (Hill, Murphy, Li‐Williams, et al. 2023), so based on these results we grouped samples into Northern NSW, Werakata NP, Gippsland and Melbourne, and evaluated only for differences in structure between the 200 and 7412 SNP datasets. To examine any difference in the identification of kin relationships, *EMIBD9* v1 (Wang 2022) was run on each dataset (with the inbreeding parameter enabled). This analysis reports kinship values between 0 (unrelated) and 0.5 (clones), where the expected value for first order (parent‐offspring or full sibling) pairs is 0.25 (Wang 2022). Pairwise kinship values ≥ 0.2 for each dataset were compared using hierarchical edge bundle plots.

## 3 Results

### 3.1 Panel Design and Development

The *Structure* analysis to separate species in the DArTseq data to facilitate panel design resulted in 329 sambar, 184 rusa and 15 hybrids (Material S1). Of the 1037 candidate loci in the first round of primer design, 492 were aligned and extended under the selected parameters; primers were designed for 300, and 252 were retained in the final panel. Following further primer design and testing, the final panel included 371 autosomal SNP loci (corresponding to DArTseq loci) and three sex SNP loci (putatively fixed gametologs), in one multiplex PCR (Table S10). Of the 371 loci, 21 were putatively fixed between sambar and rusa. Primers and amplicons ranged from 18 to 25 bp and 108 to 232 bp in length, respectively. The primers amplified to some extent in all four species in panel testing, with non‐target species clearly differentiated from sambar in a principal component analysis (PCA), but having low variation at the sequenced loci (Figure S1). The sex markers accurately identified sex in all four species in panel testing.

### 3.2 Degradation Study

After demultiplexing, 12,863,210 paired end reads matched the indexes for the project samples, with a mean per sample of 67,701. Following PEAR and GTscore, 7,221,162 reads (56%) contained both the primer and probe sequence and were on target. In total, 356 SNPs (including three sex SNPs) were genotyped in any sample, with 28.94% missing data across 190 samples prior to filtering (noting this reflects the intentional sequencing of degraded samples).

**FIGURE 1:**
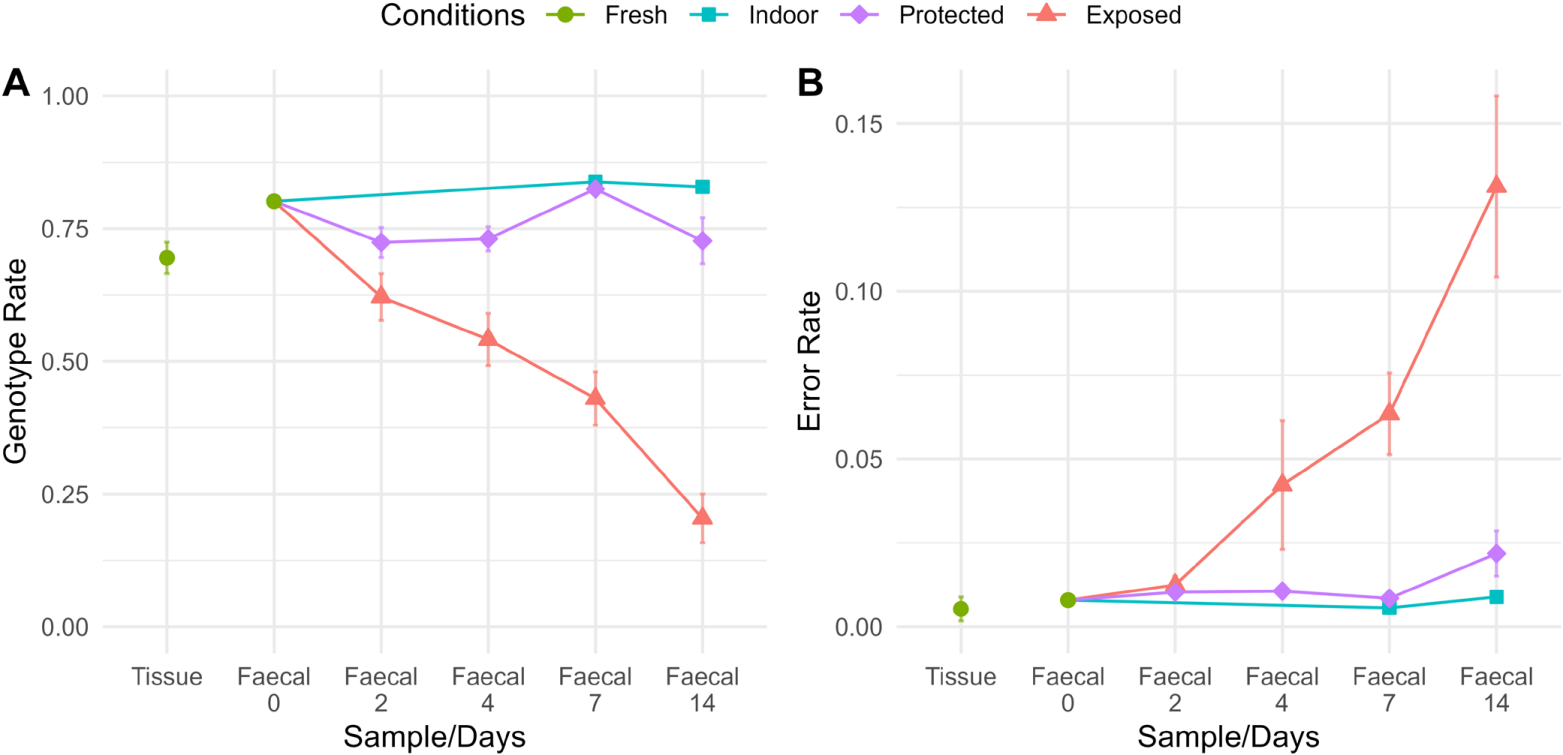
(A) Mean genotype rate (proportion of 374 loci) for fresh tissue samples and for faecal samples subjected to various conditions and amounts of time. (B) Mean error rate calculated as the proportion of alleles which differed from the representative tissue sample, considering only loci for which both samples were genotyped. Note that tissue sample error rate is calculated only from replicate samples (*n* = 2). Error bars represent standard error of the mean in both panels.

Fresh faecal samples performed equal to or better than colon tissue samples in terms of genotype rate, with an average of 0.8 (300/374 loci successfully genotyped) at Day 0 and comparatively low variance among samples (Figure 1A). For the exposed condition, there was a significant decrease in genotype rate over time relative to the indoor condition (posterior mean *β* = −0.21, 95% CI [−0.26, −0.17]; Table 2; Material S1), with the number of loci genotyped for the exposed condition reducing to, on average, 232, 202, 161, and 76 loci at 2, 4, 7, and 14 days, respectively. There was no significant change in genotype rate over time for the protected condition relative to the indoor condition (posterior mean *β* = −0.02, 95% CI [−0.06, 0.02]; Table 2; Material S3), with an average of 272 and 310 loci genotyped, respectively, at Day 14. Similarly, mean error rate was comparable between fresh faecal samples (0.008) and those of indoor and protected samples across time points (0.009 and 0.021 respectively for Day 14), with no significant difference across time for protected relative to indoor (posterior mean *β* = 0.02, 95% CI [−0.04, 0.08]; Table 2; Material S3). However, mean error rate significantly increased for exposed samples over time (posterior mean *β* = 0.18, 95% CI [0.12, 0.23]; Table 2; Material S3), with 0.014, 0.042, 0.065 and 0.131 for 2, 4, 7, and 14 days, respectively (Figure 1B). The mean error rate for replicate tissue samples (*n* = 2) was 0.005. Mean read depth and starting DNA concentrations showed patterns similar to the genotyping results, with samples protected from rain and UV being intermediate between indoor and exposed treatments (Figure S3). For colon tissue, a negative relationship was observed between measured concentration (noting samples were normalised) and genotype rate (Figure S4). Rain noticeably rehydrated faecal pellets, causing them to resemble those from Day 0 (Figure S5).

**TABLE 2:** Faecal degradation study model outputs, for genotype rate (beta regression model) and error rate (xbeta regression model) response variables.

Model	Coefficients	Estimate	Lower 95% CI	Upper 95% CI
Genotype rate	Intercept	1.33	1.05	1.62
	Day	0.02	−0.02	0.05
	ConditionsProtected	−0.16	−0.49	0.17
	ConditionsExposed	−0.28	−0.6	0.05
	Day:ConditionsProtected	−0.02	−0.06	0.02
	**Day:ConditionsExposed**	**−0.21**	**−0.26**	**−0.17**
Error rate	Intercept	−4.47	−4.96	−4.01
	Day	0.01	−0.04	0.05
	ConditionsProtected	0.08	−0.4	0.61
	ConditionsExposed	0.11	−0.35	0.61
	Day:ConditionsProtected	0.02	−0.04	0.08
	**Day:ConditionsExposed**	**0.18**	**0.12**	**0.23**

*Note:* For each model, day and conditions were fixed effects with their interaction included, and day and individual were random effects. The reference category is the indoor condition. Rows which are different from the reference category (i.e., do not include 0 in the 95% CI) are indicated in bold.

Abbreviation: CI, credible interval.

**FIGURE 2:**
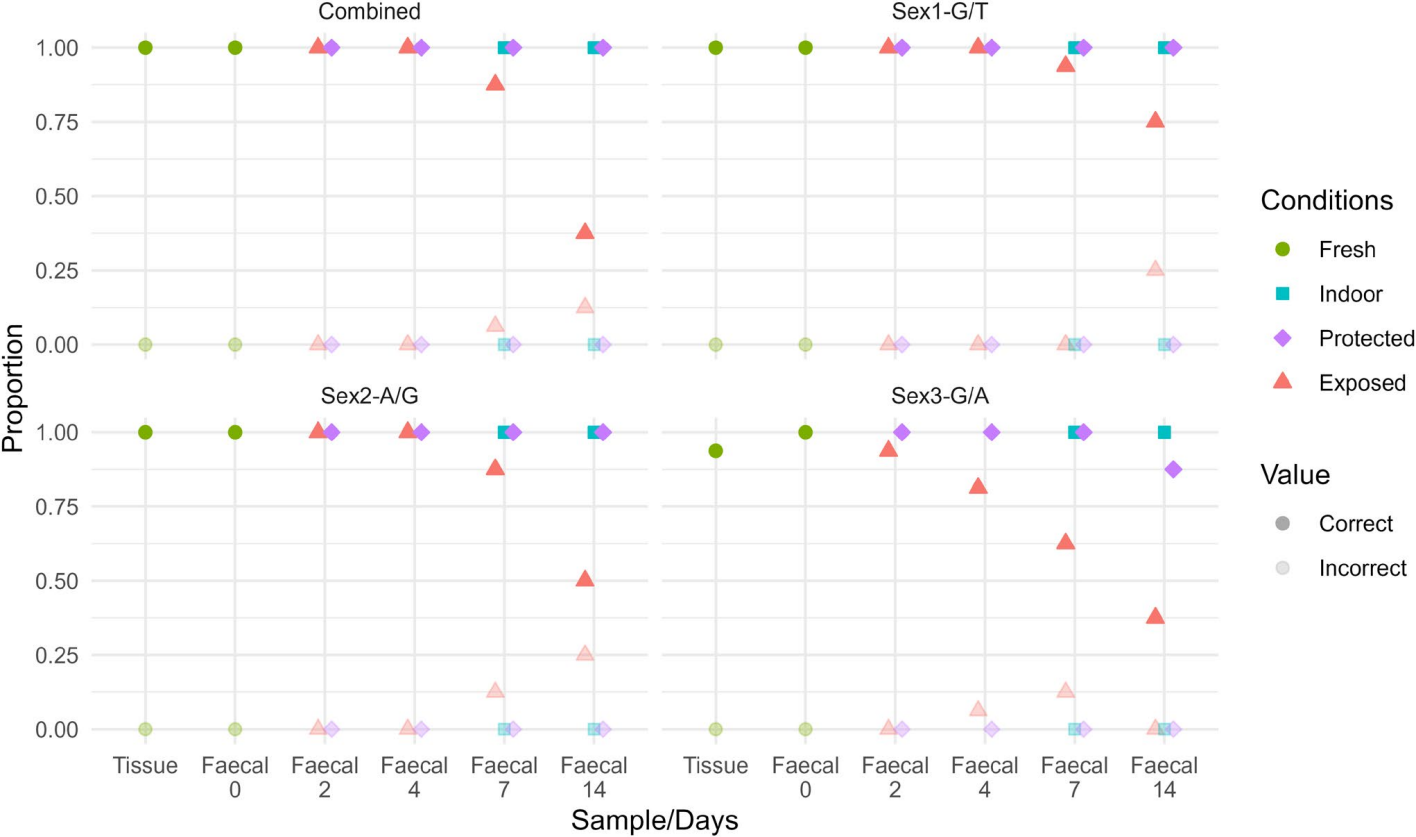
The proportion of correct and incorrect sex determination across treatments and time points for the combined approach (where genotyping required at least 2/3 loci and no disagreement), and for each sex marker individually. The proportion not genotyped (i.e., missing data) due to read depth is not explicitly shown but can be inferred from the remaining proportion.

Each of the three sex markers correctly identified sex in 100% of fresh faecal samples (*n* = 16), and 100% of indoor faecal samples across time points (*n* = 8 and *n* = 16 for Days 7 and 14, respectively) (Figure 2). A small number of tissue samples and protected faecal samples had insufficient reads to assign a genotype (i.e., missing data), but sex was never incorrectly assigned. For exposed faecal samples, however, both missing data and incorrect sex determination were observed, and increasingly so with exposure time. Under the combined approach, missing or incorrect assignments were only observed in exposed faecal samples from Days 7 and 14, and all other samples were correctly assigned. All instances of incorrect sex genotyping occurred in individuals with genotyping rate < 0.5.

**FIGURE 3:**
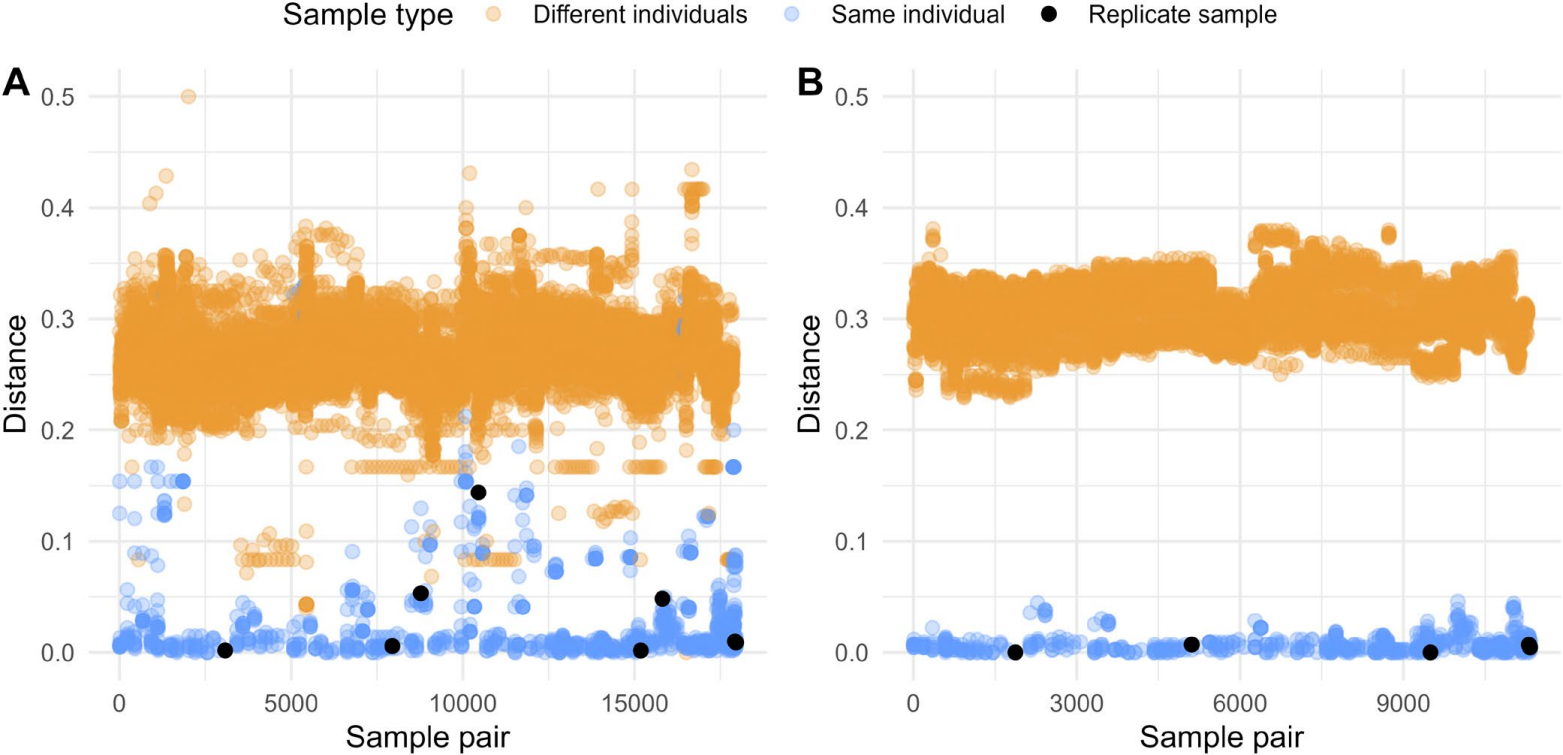
(A) Pairwise genetic distance between all 190 samples prior to filtering (356 SNPs with 28.94% missing data), with colours indicating either samples from different individuals, samples from the same individual but different time/conditions, or replicates (exact same time point and condition). Note that unfiltered data are unsuitable for individual identification and are used here only to demonstrate the importance of stringent filtering. (B) Pairwise genetic distance after filtering, resulting in 151 samples, 221 SNPs, 3.58% missing data.

Without stringent filtering, samples from degraded faecal samples produced genetic distance values which would result in incorrect individual assignment (Figure 3A). However, after applying our selected filtering thresholds (excluding samples with > 50% missing data followed by loci with > 20% missing data), genetic distances clearly differentiate comparisons of the same individual from those of different individuals, and a genetic distance threshold of 0.1 could discriminate individuals with 100% accuracy (Figure 3B). The filtered dataset retained 151 samples from 16 individuals, genotyped at 221 SNPs with 3.58% missing data. The percentage of exposed samples retained after filtering was 75% for Day 2, 63% for Day 4, 38% for Day 7, and 0% for Day 14. Few protected samples were removed across time points, with 88% retained at Day 14. Increased read depth was associated with increased genotyping rate and reduced error rate (Figure S6).

### 3.3 Panel Evaluation

Where the same individual had data passing filtering for multiple genotyping methods (amplicon and DArTseq in panel development) or sample types (colon tissue and various faecal conditions in the degradation study), genotype discordance was compared using the 200 reliable SNPs identified for panel evaluation. Genotyping discordance was extremely low between faecal and tissue samples and among faecal samples (Table 3). Discordance between DArTseq and amplicon tissue, while higher than amplicon combinations, was also suitably low for co‐analysis of data generated by different sequencing and genotype calling methods (Table 3).

**TABLE 3:** Discordance between different types of samples from the same individual that passed filtering, measured using 200 SNPs.

Sample from same individual	*n*	Mean discordance	SD	Range
Amplicon faecal – Amplicon faecal	541	0.008	0.008	0.000–0.052
Amplicon faecal – Amplicon tissue	172	0.008	0.007	0.000–0.053
Amplicon tissue – Amplicon tissue	5	0.002	0.001	0.000–0.003
Amplicon tissue – DArTseq tissue	22	0.031	0.015	0.013–0.077

*Note:* Replicates and dilutions are included.

Abbreviation: SD, standard deviation.

**FIGURE 4:**
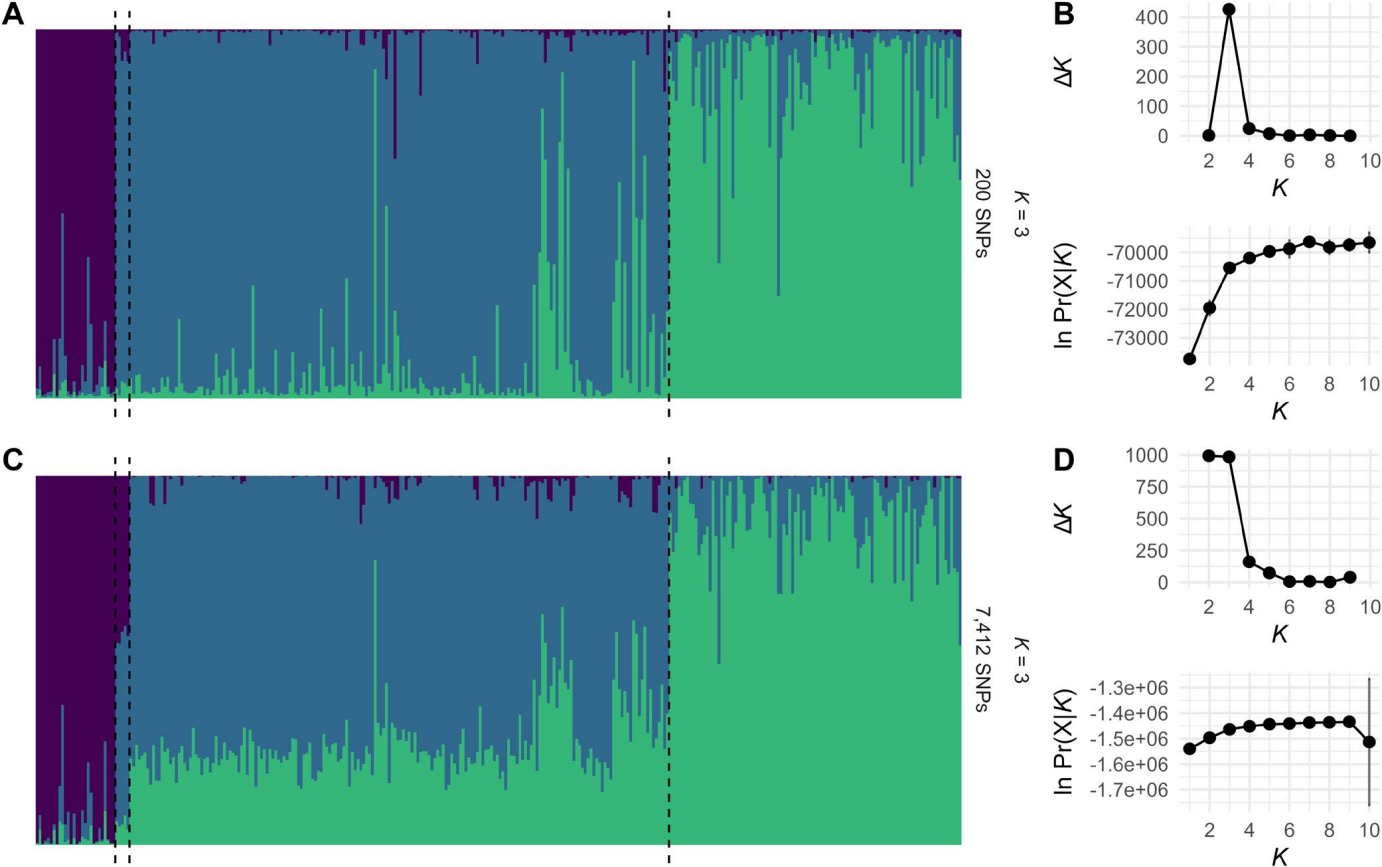
(A) *Structure* plot for *K* = 3 for the 200 SNP dataset, with each column corresponding to an individual. From left to right, the dotted lines indicate Northern NSW, Werakata NP, Gippsland, and Melbourne. (B) Evanno et al. (2005) method and ln Pr(X|*K*) method (Pritchard et al. 2000) plots for the 200 SNP dataset. (C) *Structure* plot as in (A) for the 7412 SNP dataset. (D) Plots as in (B) for the 7412 SNP dataset.

When comparing the power of the 200 reliable SNPs relative to the 7412 SNP DArTseq dataset for discriminating population structure, the same three main clusters (Northern NSW, Gippsland, and Melbourne) were identified by both datasets (Figure 4A,C). For the 200 SNP dataset, *K* = 3 was well supported by both the Δ*K* method (Evanno et al. 2005) and the ln Pr(X|*K*) method (Pritchard et al. 2000) (Figure 4B), while the best *K* was less obvious for the 7412 SNP dataset but *K* = 3 was reasonably well supported (Figure 4D). There were, however, differences between the datasets under other values of *K* (Figure S7).

**FIGURE 5:**
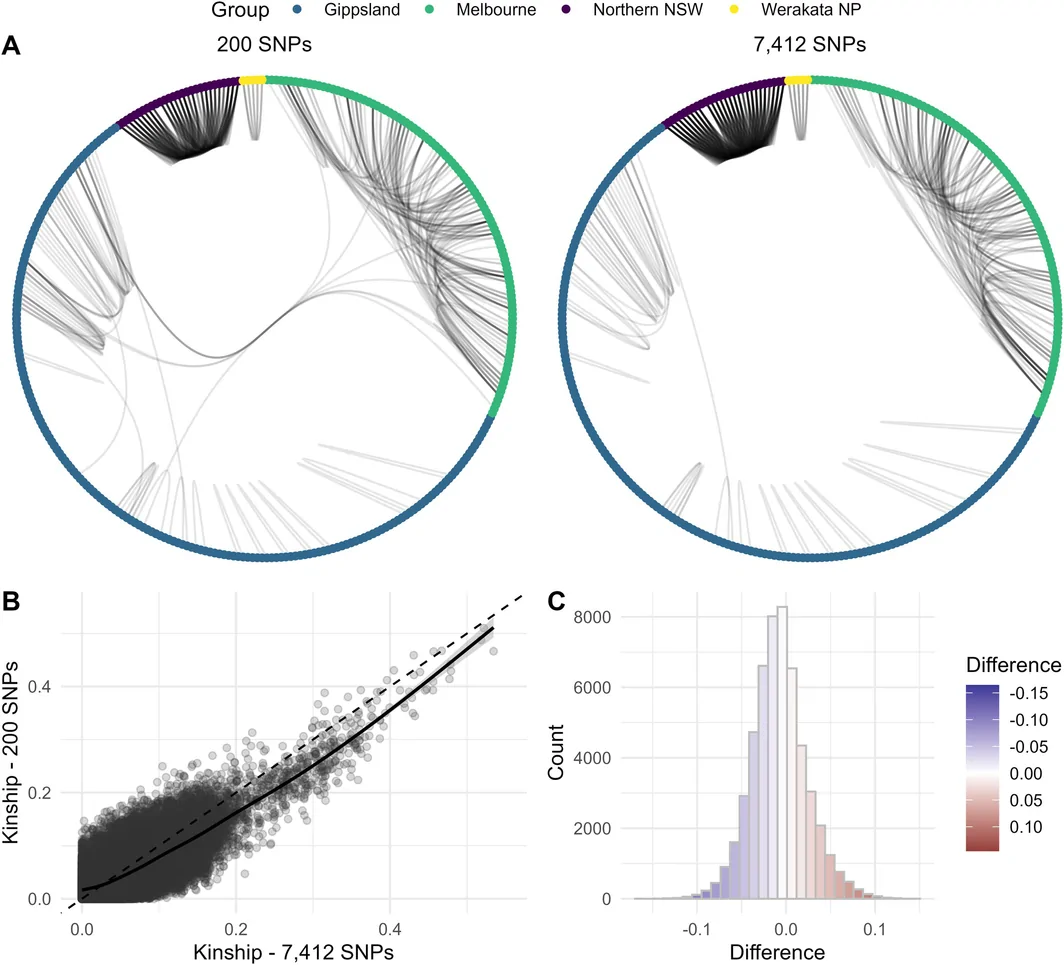
(A) Lines connecting pairs of samples with *EMIBD9* kinship values ≥ 0.2 for datasets of 200 and 7412 SNPs. (B) Relationship between kinship in the 7412 SNP dataset and the 200 SNP dataset, with the dashed line indicating *x* = *y* and the solid line a generalised additive model regression with a smooth function. (C) Difference between kinship values between datasets, where negative values indicate that the 200 SNP kinship is lower than the 7412 SNP value.

When comparing the power of the 200 and 7412 SNP datasets for identifying relatives, both datasets identified many of the same pairs of samples as the most closely related (Figure 5), but the absolute kinship values varied between datasets. Pairs with kinship values ≥ 0.2 (i.e., close relatives) in the 200 SNP dataset were also ≥ 0.2 in the 7412 SNP dataset 79.0% of the time and were ≥ 0.1 in the 7412 SNP dataset 99.6% of the time. Overall, the 200 SNP dataset enabled high‐level population structure and first‐order kinship inference, which generally represented the patterns in the 7412 SNP dataset.

## 4 Discussion

Faecal samples are a promising genetic resource for wildlife monitoring. Here, we successfully developed a GT‐seq SNP panel for sambar deer faecal samples with compatibility for co‐analysis with existing DArTseq data, providing a fit‐for‐purpose tool for cost‐effective, large‐scale genotyping of this invasive species. We demonstrated that 200 SNPs were reliably genotyped from faecal samples and suitable for inference of sex, species, individual identification, high‐level population structure and first‐order kinship. Faecal samples exposed to rain and UV were viable up to 4 days on average, while protected samples were still viable after 14 days. The degradation study is, to our knowledge, the first to examine the effects of environmental exposure on faecal DNA degradation for GT‐seq SNP genotyping.

### 4.1 Degradation

We demonstrated that while the GT‐seq approach can successfully genotype hundreds of SNP loci from deer faecal pellets, environmental exposure prior to collection is important to consider. Fresh faecal samples consistently genotyped extremely well, but our degradation study showed exposure to rainfall and UV resulted in degradation and/or loss of DNA over time, with long‐exposed pellets having fewer genotyped loci and an increased error rate. Whilst we did not separate the effects of rain and UV, with 35.6 mm rainfall recorded during the experiment, rain is expected to be the primary cause of this degradation. Although solar exposure has been associated with degradation (Cowen et al. 2022), microsatellite amplification success has been observed after long time periods (180 days) for samples exposed to UV but protected from rain (Carpenter and Dziminski 2017).

Our findings are consistent with microsatellite studies where faecal pellets exposed to natural conditions were viable for less than a week (Agetsuma‐Yanagihara et al. 2017; Brinkman et al. 2010; Piggott 2004; Woodruff et al. 2015). Interestingly, our findings differ from other SNP studies, albeit using alternative SNP genotyping approaches, where degradation effects were not detected and genotyping success was variable even among fresh faecal samples (Levengood et al. 2025; Scholz et al. 2024). However, the seminatural or simulated environmental conditions in previous SNP‐based degradation studies are not comparable to the natural conditions of the present study, with pellets placed on the ground and exposed to natural rainfall and humidity variation. These previous studies may be more comparable to our protected condition, which similarly did not show degradation. Inconsistency among studies is likely influenced by many variables including sequencing technology, study design, and DNA extraction method (i.e., surface swabbing rather than whole pellet lysis), and the relative importance of different environmental factors may also depend on geography and taxa (Lucchini et al. 2002; Maudet et al. 2004; Piggott 2004).

Most faecal samples genotyped as effectively as colon tissue in our study, adding to existing evidence that swabbing faecal pellets for intestinal epithelial cells is effective for genotyping ruminants (Davies et al. 2020; Ramón‐Laca et al. 2015). Faecal genotyping success even exceeded tissue in some cases; however, tissue samples exhibited a negative relationship between initial NanoDrop concentration and genotyping success (Figure S4), suggesting concentrations may have been inflated by residual RNA (Sanchez et al. 2015) and thus over‐diluted during normalisation. This pattern was not observed for faecal samples, but residual RNA (and other impurities) may differ between sample types and extraction protocols (noting neither included the optional RNase step). Although the faecal degradation patterns remain applicable, faecal error rates were likely slightly overestimated by considering the tissue sample as the correct genotype in pairwise comparisons. Tissue samples are generally expected to yield better results than faecal samples with GT‐seq (Arpin et al. 2024; Burgess et al. 2022).

The degradation patterns observed in our study allow for useful field sampling recommendations. For faecal pellets exposed to rain and UV, 4 days was generally the limit for most samples to be retained post filtering. However, the exposed condition in the study represents an extreme exposure scenario, with no vegetation cover and frequent rain (noting we did not separate rain and UV effects but consider rain the primary cause of degradation). Where samples can be collected from sheltered positions or in the absence of rain, we expect most faecal samples to pass filtering even up to 2 weeks. We recommend sampling when there has been no rain for several days where possible, and to prioritise either fresh‐looking pellets or those protected by vegetation. Avoiding sampling directly after rain will prevent the collection of pellets which have been rehydrated, which, as observed here (Figure S5) and reported previously (Agetsuma‐Yanagihara et al. 2017), makes pellets appear deceptively fresh. While moisture and visible mucus indicate very fresh pellets, those which are hard and dry should not be discounted. Desiccation is a DNA preservation method (Allison et al. 2021; Frantzen et al. 1998; Piggott and Taylor 2003) and DNA may be well preserved if rain exposure has been minimal. As pellet age in the field is unknown and estimation is subjective, investigation of whether visual characteristics of pellets (e.g., colour, texture) can predict genotyping success is warranted, an approach which has varied among species previously (Piggott 2004).

### 4.2 Panel Suitability

By comparing faecal samples from known individuals under different environmental exposure, we confirmed that our SNP panel had sufficient resolution to clearly differentiate sambar individuals based on genetic distance. However, with inadequate filtering, long exposed samples could generate incorrect conclusions, either via different individuals appearing similar due to insufficient loci, or the same individual appearing different due to genotyping error. Both factors are associated with reduced read depth (Figure S6) and addressed with stringent filtering. Here, filtering samples based on 50% genotyping rate (followed by 80% locus genotyping) resulted in 100% correct assignment of individuals, and we recommend this approach when applying the panel in field samples to ensure sufficient data quality for individual assignment and comparability between applications. In both the development and application of panels intended for faecal samples, we recommend thoroughly establishing the appropriate filtering and identification thresholds by sequencing faecal samples of known shared identity (replicates). Given the 100% individual assignment accuracy (provided strict filtering is maintained), our panel will be extremely useful for management applications such as population size estimates (Bellemain et al. 2005; Li et al. 2020), monitoring the outcomes of population control measures (Piggott et al. 2008; White et al. 2025), and even tracking individual movement (Eriksson et al. 2020).

Target species verification is an important consideration in non‐invasive monitoring, and faecal pellet appearance cannot reliably differentiate the deer species which co‐occur in southeastern Australia. Our panel consistently separated sambar from non‐target deer species in a PCA and had sufficient resolution for identifying sambar‐rusa hybrids (Figure S1). Eriksson et al. (2020) similarly included SNPs to differentiate coyotes from wolves in their amplicon panel (with initial metabarcoding to exclude other carnivores), while Ahrens et al. (2024) included diagnostic mtDNA markers for distinguishing bobcats from other carnivores in their microfluidic SNP panel. Given that hybridisation between sambar and rusa poses a management issue (Hill, Murphy, Li‐Williams, et al. 2023), and that the spread of different deer species to new sites may be otherwise difficult to detect in low densities, prompt identification of these events may be further benefits of monitoring using the panel.

Whilst sex can often be documented at collection for invasive sampling methods (depending on species), accurate genetic sex determination is crucial for non‐invasive methods. The three sex markers developed in this study correctly assigned sex for all deer sequenced in panel development (*n* = 38) and the degradation study (*n* = 16). Given these comprised four species, results suggest the designed markers are indeed fixed XY gametologs suitable for genetic sex determination across deer species. This is supported by the consistency among numerous Artiodactyla taxa during in silico testing (Tables S2–S9). Incorrect sex determination was occasionally reported in exposed samples after Day 4 (under the combined approach), but, like the autosomal SNP genotyping, stringent filtering can ensure accuracy. Along with excellent multiplex genotyping success, the developed sex markers allowed for streamlined analysis compared to length‐based sex markers and are applicable in related taxa.

Genotyping agreement between samples from the same individual is important both for individual identification and for combining data generated by different methods. Encouragingly, discordance between samples from the same individual using the panel was extremely low (< 1%), both between faecal and tissue samples and among faecal samples (which passed filtering). Other studies which compared colon faeces and paired tissue using GT‐seq have reported 6.8% (Hayward et al. 2022) and 0.74% discordance (Burgess et al. 2022), with the latter comparable to the 0.8% discordance between tissue and faeces in the present study. Discordance with DArTseq data, although higher (3%), still confirmed that datasets generated using the panel can be co‐analysed with existing data. This level of discordance is comparable with that reported for paired GT‐seq and RADseq data: 2.57% by Schmidt et al. (2020), 4% or 7% by Garrett et al. (2024), and 4.7% by Arpin et al. (2024), often attributed to genotype calling differences. The overall error rate for the DArTseq method has also been reported as 4% (Garavito et al. 2016). To our knowledge, the only other targeted faecal approach developed from DArTseq SNPs involved MassARRAY panels for monitoring bats (Thavornkanlapachai et al. 2024), however, discordance between the methods was not examined.

Analysis of population structure and kinship is essential for understanding management units and dispersal in invasive species management (Le Roux and Wieczorek 2009), and our results demonstrate that the overall sambar deer population structure found using DArTseq can be detected with the 200 SNPs our panel can reliably genotype. However, regardless of the number of markers, population structure and kinship analyses in sambar deer are impacted by high background relatedness (Hill, Murphy, Li‐Williams, et al. 2023; Hill et al. unpublished data). In the 7412 SNP dataset, kinship values higher than 0.3 (while still distinct genotypes) were frequent. This suggests breeding among related individuals, which inhibits exact relationship classification. For measuring pairwise kinship, the 200 SNP and 7412 SNP datasets were correlated but variable, with lower resolution in the panel. The level of kinship accuracy observed is consistent with the simulations performed by Burgess et al. (2022), and is still a highly useful addition to monitoring. We do however recommend that only relationships with relatively high pairwise kinship (≥ 0.2) should be considered as true relatives, acknowledging that some close relative pairs are likely to be missed (false negatives), and some relationships would be overestimated, but that false positives would be extremely rare. As our panel has sufficient resolution for high‐level population structure and first‐order kinship, incorporating faecal genotyping with existing DArTseq data will enhance understanding of dispersal and management units, including identifying potential sources for any newly established populations.

The ability to genotype faecal samples allows for targeted sample collection and repeated sampling of living individuals, but these benefits must be weighed against the costs compared to existing methods. Where faecal samples replace invasive handling of wildlife and the associated permits, equipment and labour involved, costs for sample collection would be greatly reduced. Using the protocol we recommend based on our study, the cost of consumables to genotype a plate of 96 samples is estimated at $36 AUD per sample, with cost‐effectiveness increasing with scale (Material S4). Processing one plate from DNA extraction to sequencing can be performed in 1–2 weeks. For an established molecular laboratory with access to in‐house sequencing, costs could therefore be comparable to existing methods. Genotyping costs could be further reduced with fewer loci, as primers comprise approximately half the cost for one‐off applications, and the maximum observed genotyping rate (0.88) suggests room for improvement. However, in panel testing, the genotyping rate was similar across various multiplex sizes. Due to the disproportionate amplification involved in multiplex PCR, and a different species' genome being used for primer design, efforts to further optimise the panel were deemed unnecessary in the present study. For new panel development, the trade‐off between number of SNPs, genotyping rate, and optimisation sequencing should be considered, and tailored to the specific requirements and intended future use, whether small‐scale applications or truly ‘Genotyping‐in‐Thousands by sequencing’ with the GT‐seq method.

### 4.3 Concluding Remarks

Non‐invasive samples are an appealing, cost‐effective and ethical option for wildlife monitoring, but challenging to genotype. This study provides the first demonstration of the impacts of faecal degradation on SNP amplicon panel genotyping. The results offer a pathway for sampling faecal pellets that are most likely to yield suitable DNA and demonstrate that faecal‐derived genotypes can provide sufficient resolution for population genetic analysis and can be combined with existing DArTseq data. This facilitates targeted, non‐invasive studies which are compatible with but not reliant on traditional sampling methods, an approach with broad monitoring applications across both native and invasive species. Potential applications include estimating population size via genetic mark‐recapture over repeat sampling visits (Bellemain et al. 2005; Li et al. 2020); comparing populations before and after management actions to evaluate success (Piggott et al. 2008; Smith et al. 2009; White et al. 2025); and using individual movement tracking (Eriksson et al. 2020) or relatedness between individuals (Norman and Spong 2015) to understand dispersal, management units, or the source of new individuals or populations. These insights for the development and integration of faecal DNA genotyping with existing techniques enable innovative questions to be addressed with non‐invasive genetic wildlife research.

## Author Contributions

**Tamandra H. D'Ombrain:** formal analysis (lead), funding acquisition (lead), investigation (lead), methodology (lead), project administration (equal), resources (equal), visualization (lead), writing – original draft (lead), writing – review and editing (equal). **Katherine A. Harrisson:** methodology (equal), supervision (equal), writing – review and editing (equal). **Carlo Pacioni:** conceptualization (equal), methodology (equal), supervision (equal), writing – review and editing (equal). **Erin Hill:** methodology (equal), resources (supporting), writing – review and editing (supporting). **Nicholas P. Murphy:** conceptualization (equal), methodology (equal), project administration (equal), resources (equal), supervision (lead), writing – review and editing (equal).

## Funding

This work was supported by Holsworth Wildlife Research Endowment. La Trobe University.

## Conflicts of Interest

The authors declare no conflicts of interest.

## Supporting information


**Figure S1:** PCA of different species.
**Figure S2:** Pilot study.
**Figure S3:** Read depth and concentration over time.
**Figure S4:** Relationship between NanoDrop concentration and genotype rate.
**Figure S5:** Appearance of faecal pellets.
**Figure S6:** Read depth and genotyping relationship.
**Figure S7:** Structure for different *K*.
**Material S1:** Structure for species identification.
**Material S2:** Individual assignment simulation.
**Material S3:** Degradation modelling.
**Material S4:** Costs.
**Table S1:** Sex primers.

**Table S2:** SNP gametolog sex markers developed for this study, with sequences used to differentiate *X* and *Y*.
**Table S3:** Chromosomes used for in silico testing of sex markers.
**Table S4:** Broadest in silico testing where Sex1‐G_T marker appropriate for sex determination – Artiodactyla.
**Table S5:** In silico testing where Sex1‐G_T marker not appropriate for sex determination – Mammalia.
**Table S6:** Broadest in silico testing where Sex2‐A_G marker appropriate for sex determination – Ruminantia.
**Table S7:** In silico testing where Sex2‐A_G marker not appropriate for sex determination – Artiodactyla.
**Table S8:** Broadest in silico testing where Sex3‐G_A marker appropriate for sex determination – Cervidae.
**Table S9:** In‐silico testing where Sex3‐G_A marker not appropriate for sex determination – Ruminantia.
**Table S10:** Primers for 374 panel loci, including full primer sequences ordered with Illumina adapter sequences indicated in lowercase.
**Table S11:** Corresponding 69bp DArT sequences.
**Table S12:** GTscore probefile used for genotyping.

## Data Availability

Raw Illumina reads are available from the NCBI Sequence Read Archive (BioProject: PRJNA1421047). Sex primer information is available in Tables [Link], [Link]. Panel primers, target DArT tags and genotyping probe file are available in Tables S10–S12. Scripts and metadata are available from Figshare (https://doi.org/10.26181/31302583).
