# Supplementary material for: Development of a SNP Amplicon Panel for Invasive Deer Faecal Pellets and the Effects of Environmental Exposure on Genotyping Success
Source: Ecol Evol. 2026 Sep 21;16(9):e74388. doi: 10.1002/ece3.74388 (PMC13593369; doi:10.1002/ece3.74388)
Supplement: Supplementary file 1 [file ECE3-16-e74388-s002.pdf]

Supporting Information for:

Development of a SNP amplicon panel for invasive deer faecal pellets and the effects of environmental exposure on genotyping success

Tamandra H. D'Ombrain[1], Katherine A. Harrisson[1], Carlo Pacioni[2], Erin Hill[3], and Nicholas P. Murphy[1]

[1]Department of Ecological, Plant and Animal Sciences, La Trobe University, Bundoora 3086, Victoria, Australia
[2]Department of Energy, Environment and Climate Action, Arthur Rylah Institute for Environmental Research, Heidelberg 3084, Victoria, Australia
[3]Health and Biosecurity, CSIRO, Black Mountain 2601, Australian Capital Territory, Australia

Table of Contents

Supplementary Material S1 – Structure for species identification 2
Supplementary Material S2 – Individual assignment simulations 3
Supplementary Material S3 – Degradation modelling 4
Supplementary Material S4 – Costs 6
Table S1 – Sex primers 8
Figure S1 – PCA of different species 8
Figure S2 – Pilot study 9
Figure S3 – Read depth and concentration over time 9
Figure S4 – Relationship between NanoDrop concentration and genotype rate 10
Figure S5 – Appearance of faecal pellets 10
Figure S6 – Read depth and genotyping relationship 11
Figure S7 – Structure for different K 11

Supplementary Material S1 – Structure for species identification

The initial dataset contained 739 co-analysed samples from sambar, rusa, fallow deer and hybrids. The samples are described in the associated publications (Hill, Murphy, Li-Williams, et al., 2023; Hill et al., in prep.; Li-Williams et al., 2023; White et al., 2026), however, the exact subset of DArTseq data used as the basis of our panel development were not identical to one particular study. Therefore, species assignment for our specific dataset was required. Following removal of fallow deer (based on PCA grouping) and samples with genetic similarity indicating replicates or identical twins, 532 individuals remained. The data were filtered using the dartR package (Gruber et al., 2018; Mijangos et al., 2022) by reproducibility (threshold = 1), locus callrate (threshold = 0.98), individual callrate (threshold = 0.75 removing 4 individuals), minor allele frequency (threshold = 0.01), and secondaries ("best" method), with any monomorphic loci removed, resulting in 528 samples and 7,341 loci. The *Structure* v2.3.4 (Pritchard et al., 2000) analysis applied to this dataset to distinguish between sambar and rusa included 20 runs of K=2, with 100,000 burnin and 500,000 MCMC iterations. The results of the *Structure* Qmatrix were merged across runs, giving each sample a proportional value for each cluster with extremes corresponding to the purest individuals of a species. This information was used as a genetic basis to distinguish sambar from rusa deer. Samples scoring between 0.9-1 for the sambar cluster were considered "pure" sambar.

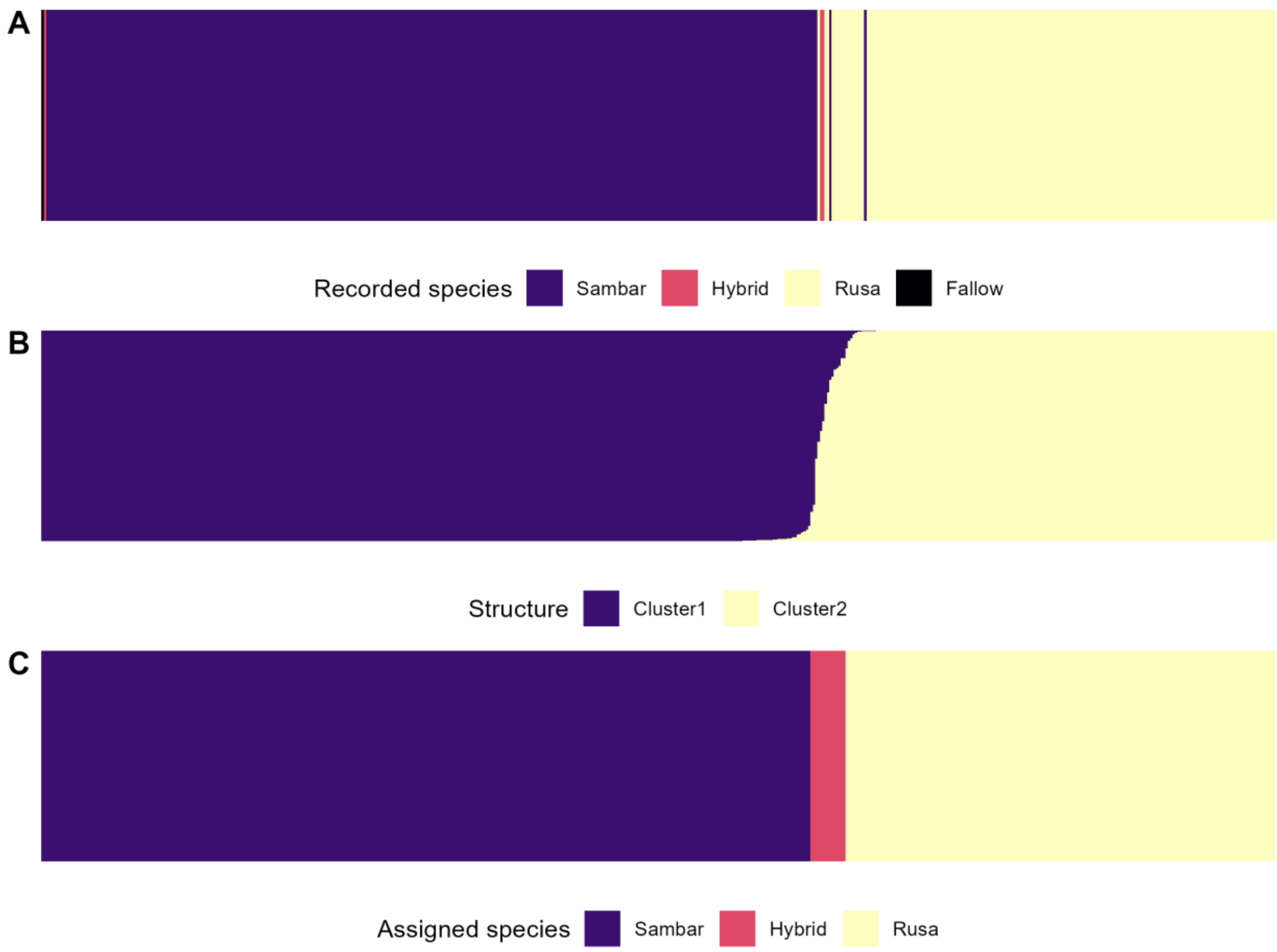

Supplementary Material S1 Figure 1. Plots with each bar representing an individual. (A) Species which was recorded upon sample collection. (B) *Structure* analysis showing proportional allocation of each individual to genetic clusters corresponding to sambar and rusa. (C) Genetic species assignment used, where >=0.9 assignment to a cluster was required for species assignment, and lower values were considered hybrids.

Supplementary Material S2 – Individual assignment simulations

While metrics such as probability of identity (PID) aim to ensure sufficient marker variability to prevent identical multilocus genotypes from different individuals (Waits et al., 2001), in large SNP datasets, even samples from the same individual are rarely identical. Instead, individual assignment relies on samples from the same individual having exceptionally low genetic distance values (or high relatedness) relative to pairwise comparisons between different individuals. Accurate individual assignment in introduced sambar deer must consider low population-level genetic variation (Rollins et al., 2023) and high background relatedness (Hill et al., in prep.), as well as increased missing data and error due to genotyping faecal samples.

Simulations began with the sambar DArTseq data used for panel design, randomly subset to 100 individuals. Datasets were created with MAF thresholds 0.01, 0.1 and 0.2, then each of these with 50, 100, 200 and 400 randomly selected SNPs. Each dataset had 20 clones added (by duplicating 20 individuals), missing data standardised to 5%, then allele errors randomly generated according to error rates 0.01, 0.05 and 0.1. The resulting simulations suggest that individual identification is inhibited by increasing error rate, but higher MAF and number of SNPs help to increase separation between groups and reduce variation. However, note that error rate would not affect a dataset uniformly but rather vary with sample quality.

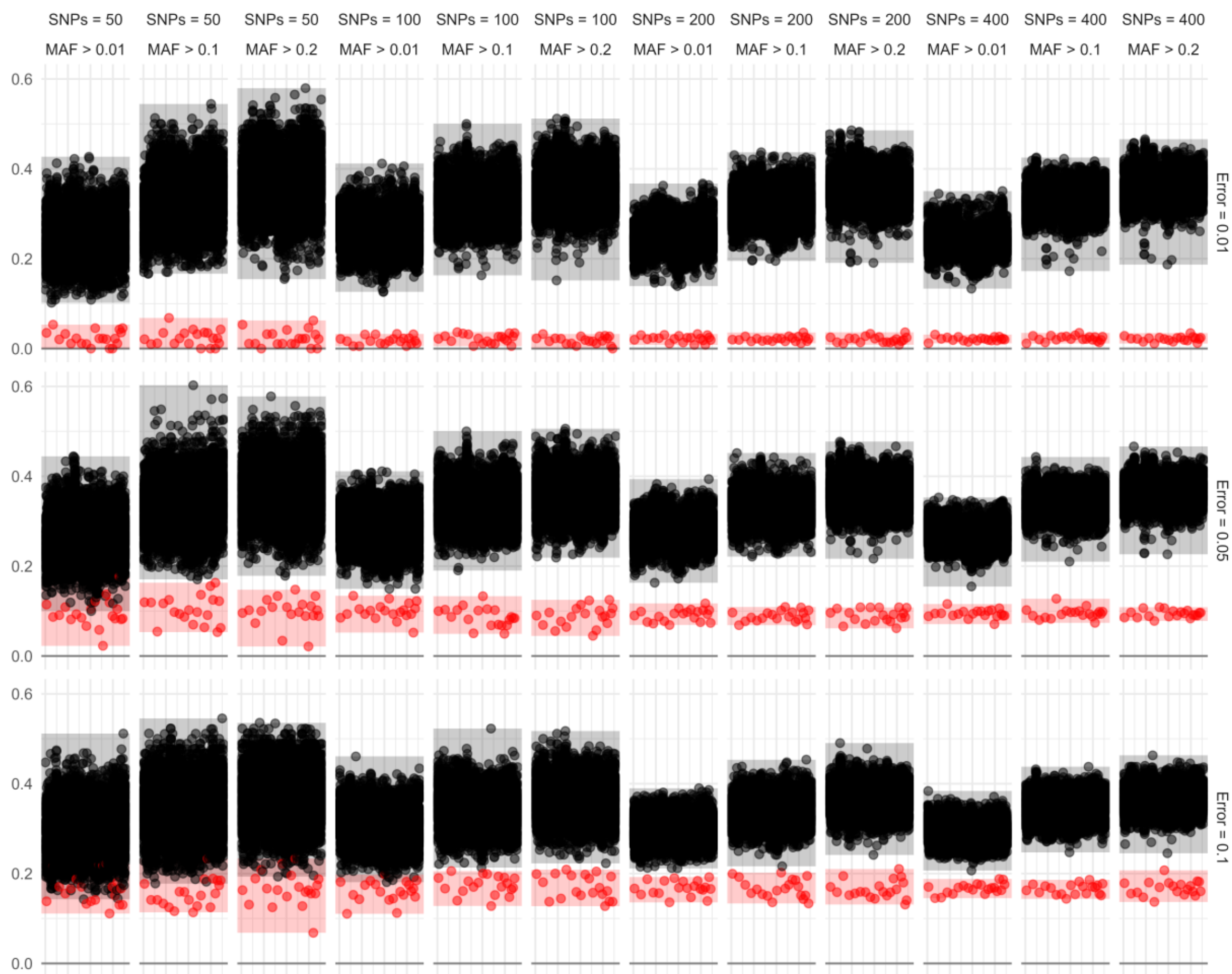

Supplementary Material S2 Figure 1. Pairwise genetic distance comparisons for 100 unique individuals and 20 replicates, with varying minor allele frequency thresholds (0.01, 0.1 and 0.2), number of SNPs (50, 100, 200 and 400), and error rate (0.01, 0.05 and 0.1). Red points represent pairwise comparisons of the same individual (by duplicating 20 individuals prior to generating errors), while black points are different individuals.

Supplementary Material S3 – Degradation modelling

Genotype rate

Supplementary Material S3 Table 1. Model comparisons using 'loo' for GenotypeRate, with the smallest values for elpd_diff and se_diff corresponding to the best fit and therefore the selected model. Formula is in brms package syntax.

Model formula	elpd_diff	se_diff
GenotypeRate ~ Day * Conditions + (1 + Day | Individual)	0	0
GenotypeRate ~ Day + Conditions + (1 + Day | Individual)	-50.8	11.2

Final model information:
Family: beta, Links: mu = logit, Formula: GenotypeRate ~ Day * Conditions + (1 + Day | Individual), Draws: 4 chains, each with iter = 2000; warmup = 1000; thin = 1; total post-warmup draws = 4000

Supplementary Material S3 Table 2. Detailed outputs for the beta regression model with genotype rate as the response variable and day and condition as fixed effects. Day and individual were included as random effects to account for repeated sampling of individuals across time. The reference category is the indoor treatment.

Coefficients	Estimate	Est.Error	l-95% CI	u-95% CI	R-hat	Bulk_ESS	Tail_ESS
Intercept	1.33	0.15	1.05	1.62	1	2946	2747
Day	0.02	0.02	-0.02	0.05	1	2857	2755
ConditionsProtected	-0.16	0.17	-0.49	0.17	1	3255	2947
ConditionsExposed	-0.28	0.17	-0.6	0.05	1	3267	2958
Day:ConditionsProtected	-0.02	0.02	-0.06	0.02	1	3427	3288
Day:ConditionsExposed	-0.21	0.02	-0.26	-0.17	1	3299	3256

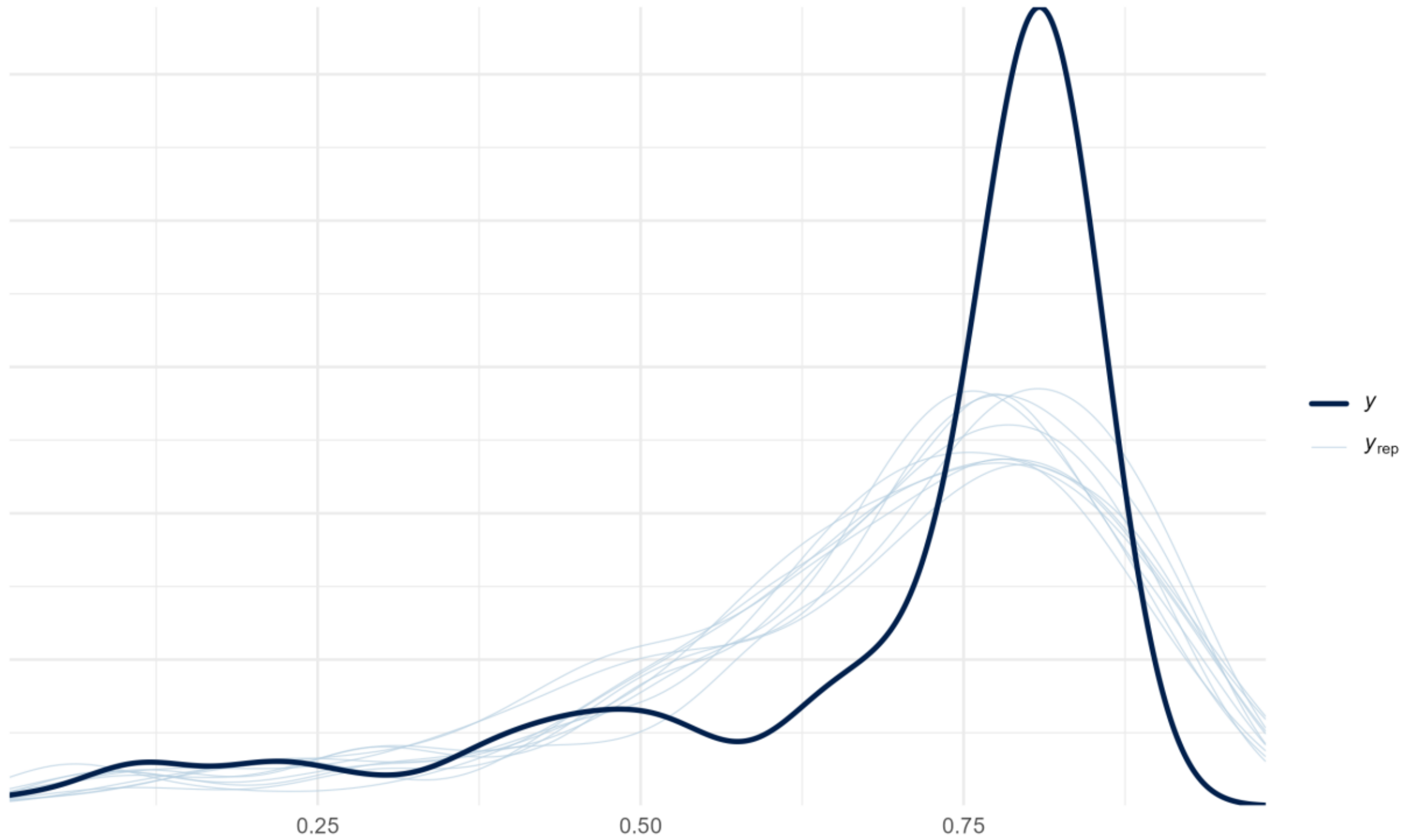

Supplementary Material S3 Figure 1. Posterior predictive check for genotype rate model.

Error rate

Supplementary Material S3 Table 3. Model comparisons using 'loo' for ErrorRate, with the smallest values for elpd_diff and se_diff corresponding to the best fit and therefore the selected model. Formula is in brms package syntax.

Model formula	elpd_diff	se_diff
ErrorRate ~ Day * Conditions + (1 + Day | Individual)	0	0
ErrorRate ~ Day + Conditions + (1 + Day | Individual)	-26	12.5

Final model information:

Family: xbeta, Links: mu = logit, Formula: ErrorRate ~ Day * Conditions + (1 + Day | Individual), Draws: 4 chains, each with iter = 2000; warmup = 1000; thin = 1; total post-warmup draws = 4000

Supplementary Material S3 Table 4. Detailed outputs for the xbeta regression model with error rate as the response variable, and day and condition as fixed effects. Day and individual were included as random effects to account for repeated sampling of individuals across time. The reference category is the indoor treatment.

Coefficients	Estimate	Est.Error	l-95% CI	u-95% CI	R-hat	Bulk_ESS	Tail_ESS
Intercept	-4.47	0.24	-4.96	-4.01	1	1899	2407
Day	0.01	0.02	-0.04	0.05	1	1572	2474
ConditionsProtected	0.08	0.25	-0.4	0.61	1	1878	2508
ConditionsExposed	0.11	0.24	-0.35	0.61	1	1887	2795
Day:ConditionsProtected	0.02	0.03	-0.04	0.08	1	1527	2459
Day:ConditionsExposed	0.18	0.03	0.12	0.23	1	1496	2199

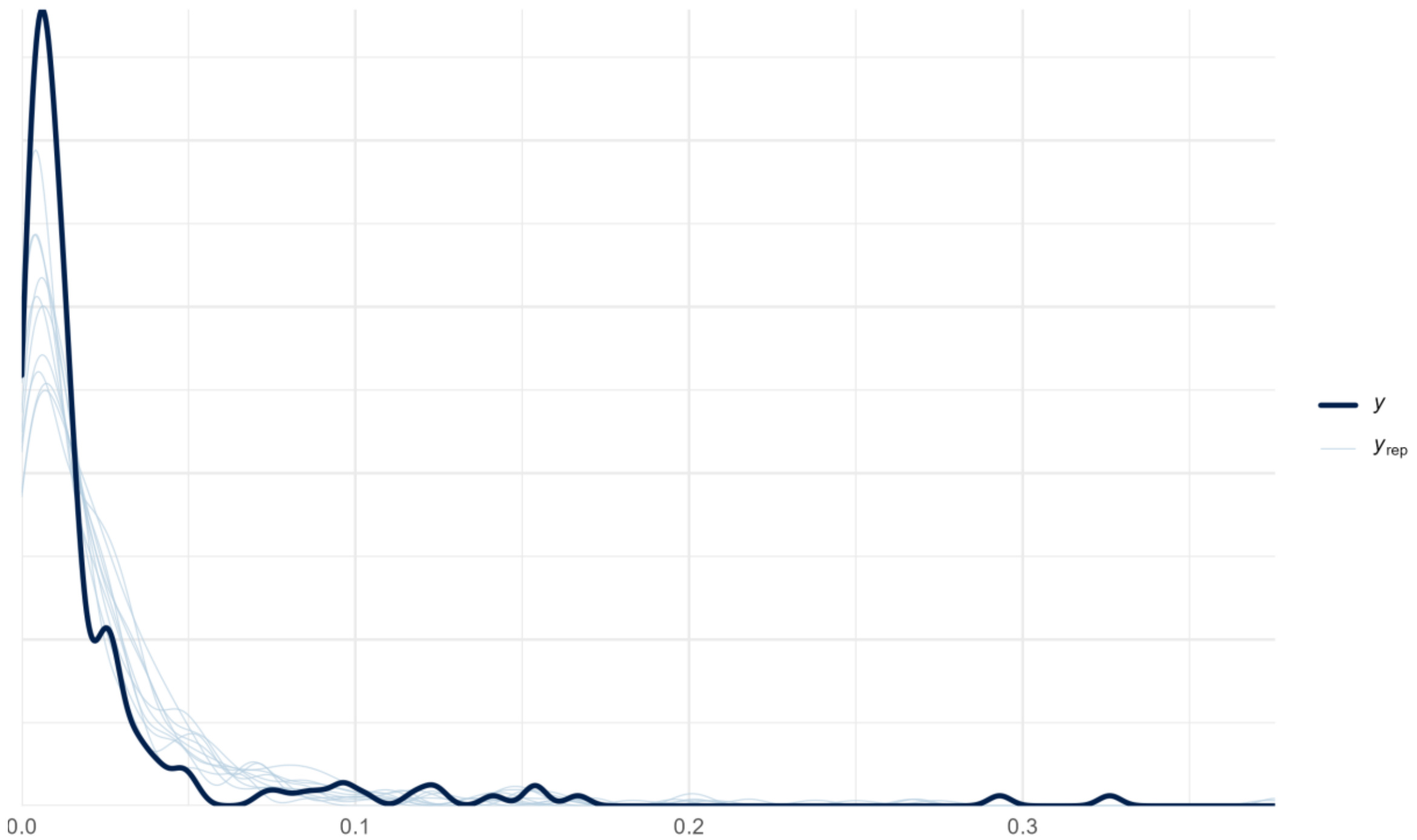

Supplementary Material S3 Figure 2. Posterior predictive check for error rate model.

Supplementary Material S4 – Costs

Supplementary Material S4 Table 1. Cost of consumables to sequence one plate of 96 faecal samples using the final recommended method with a small MiSeq kit. Calculations are based on September 2024 AUD prices accessible to a university molecular lab and items which are not project-specific are calculated based on amount required assuming products are bought in bulk. Processing from DNA extraction to sequencing involves 1–2 weeks labour. Calculations include purchasing small amounts of pre-pooled primers (up to 200 rxns at the ratios used) for a single application of the method, but purchasing primers individually and preparing pools in-house becomes more cost-effective for large scale sequencing. Costs are increasingly efficient when processing multiple plates on a larger kit (e.g. four plates can be sequenced for ~$8,800; $23/sample). Prior to implementing a newly designed panel, 2–3 sequencing runs may be required to test and optimise the primer multiplex.

Step/product	Cost
DNA extraction x 96 swabs	$ 385.99
Qubit* quantification + normalise (optional)	$ 134.98
Primers (2 x 187 loci oPool)	$ 1,748.47
PCR1	$ 69.76
Gel (optional)	$ 4.26
Magnetic bead clean	$ 40.54
PCR2	$ 46.66
Gel (optional)	$ 4.26
Magnetic bead clean	$ 40.54
Gel (optional)	$ 4.26
ClarioSTAR/Qubit quantification + normalise	$ 142.62
Pool + TapeStation* fragment length analysis + sequence	$ 81.67
MiSeq Reagent Micro Kit v2 (300 cycles - 2x150)	$ 770.40
**Total**	**$ 3,474.40**
**Per sample**	**$ 36.19**

* = not the procedure used in the present study, but recommended in future applications

Supplementary Material S4 Table 2. Consumables required and costs used in calculations. Small volumes of common laboratory reagents (e.g. ethanol, NaOH) are also needed. Required equipment includes a thermocycler, magnetic plate and MiSeq along with standard laboratory equipment for temperature control, measuring, mixing, pipetting and gel electrophoresis.

Process	Item	Price	Amount	Unit	Cost/Unit
General	Pipette tips filter average	$ 0.092	1	each	$ 0.092
	Pipette tips unfiltered average	$ 0.014	1	each	$ 0.014
	Gloves	$ 9.30	250	each	$ 0.037
	1.5 mL microcentrifuge tubes	$ 8.20	500	each	$ 0.016
	2 mL microcentrifuge tubes	$ 60.20	2000	each	$ 0.030
	15 mL tubes	$ 228.00	1000	each	$ 0.228
	50 mL tubes	$ 111.00	500	each	$ 0.222
	PCR plates	$ 439.00	100	each	$ 4.390
	PCR strip lids	$ 37.00	125	each	$ 0.296
DNA extraction	Buffer ATL	$ 149.40	200	mL	$ 0.747
	Buffer AL	$ 149.40	264	mL	$ 0.566
	Longmire buffer*	$ 41.130	1000	mL	$ 0.041
	Proteinase K	$ 821.00	10000	µL	$ 0.082
	Swabs	$ 36.80	100	each	$ 0.368
	Scalpel blades	$ 17.80	100	each	$ 0.178
	Spin columns	$ 106.00	100	each	$ 1.060
	RNase A	$ 501.30	2500	µL	$ 0.201
	Buffer AW1	$ 150.30	500	mL	$ 0.301
	Buffer AW2	$ 149.40	500	mL	$ 0.299
PCR	Multiplex PCR Master Mix	$ 495.00	5000	µL	$ 0.099
	GoTaq Green Master Mix	$ 634.58	25000	µL	$ 0.025
	Primer oPools	$ 1,748.47	200	uL	$ 8.742
	Illumina index primers*	$ 0.006	1	µL	$ 0.006
Quantification	Plate-reader plates	$ 323.00	100	each	$ 3.230
	Qubit tubes	$ 34.05	1000	each	$ 0.034
	Qubit HS reagents	$ 537.03	500	rxns	$ 1.074
Gel electrophoresis	Agarose	$ 877.79	500	g	$ 1.756
	DNA ladder	$ 384.37	3750	µL	$ 0.102
	GelRed	$ 228.44	500	µL	$ 0.457
	TBE	$ 211.20	40000	mL	$ 0.005
Magnetic bead clean	Magnetic bead solution*	$ 58.610	50000	µL	$ 0.001
	Round bottom plates	$ 157.00	100	each	$ 1.570
Sequencing	PhiX	$ 274.50	10	µL	$ 27.450
	MiSeq Reagent Micro Kit v2	$ 770.40	1	each	$ 770.400

* = reagent prepared in-house.

Table S1 – Sex primers

Table S1. Sex primer combinations tested in panel development. Only the three primer sets designed in this study were retained in the final panel. Oligos were ordered with Illumina adapter sequences preceding the primer.

	Gene/region	Direction	Name	Primer	Type	Reference
1	AMELX/AMELY	Forward<br>Reverse	KY1<br>KY2	5'-gcccagcagcccttccag-3'<br>5'-tggccaagcttccagaggca-3'	Length	Yamauchi et al. (2000)
2	AMELX/AMELY	Forward<br>Reverse	KY1<br>-	5'-gcccagcagcccttccag-3'<br>5'-gctggccaagcttccaga-3'	Length	Yamauchi et al. (2000)<br>Gurgul et al. (2009)
3	ZFX/ZFY	Forward X<br>Forward Y<br>Reverse	BJSexX-F<br>BJSexY-F<br>BJSex-R1	5'-agaccaggttcccttcat-3'<br>5'-tctggaccaaattcactttat-3'<br>5'-gggccaataattattgctaag-3'	Length	Kim et al. (2009)
4	UTX/UTY (KDM6A/KDM6C)	Forward<br>Reverse	Sex1-G/T F<br>Sex1-G/T R	5'-cccaacttctcacctgctgt-3'<br>5'-aggctcagttgtgtaaccttcc-3'	SNP	This study
5	ZFX/ZFY	Forward<br>Reverse	Sex2-A/G F<br>Sex2-A/G R	5'-aacccttgttcagctgtctca-3'<br>5'-cacctggagagccacaagc-3'	SNP	This study
6	-	Forward<br>Reverse	Sex3-G/A F<br>Sex3-G/A R	5'-ggccattctagttgccattaaaag-3'<br>5'-aacccagttcaaaatgtctcttac-3'	SNP	This study

Figure S1 – PCA of different species

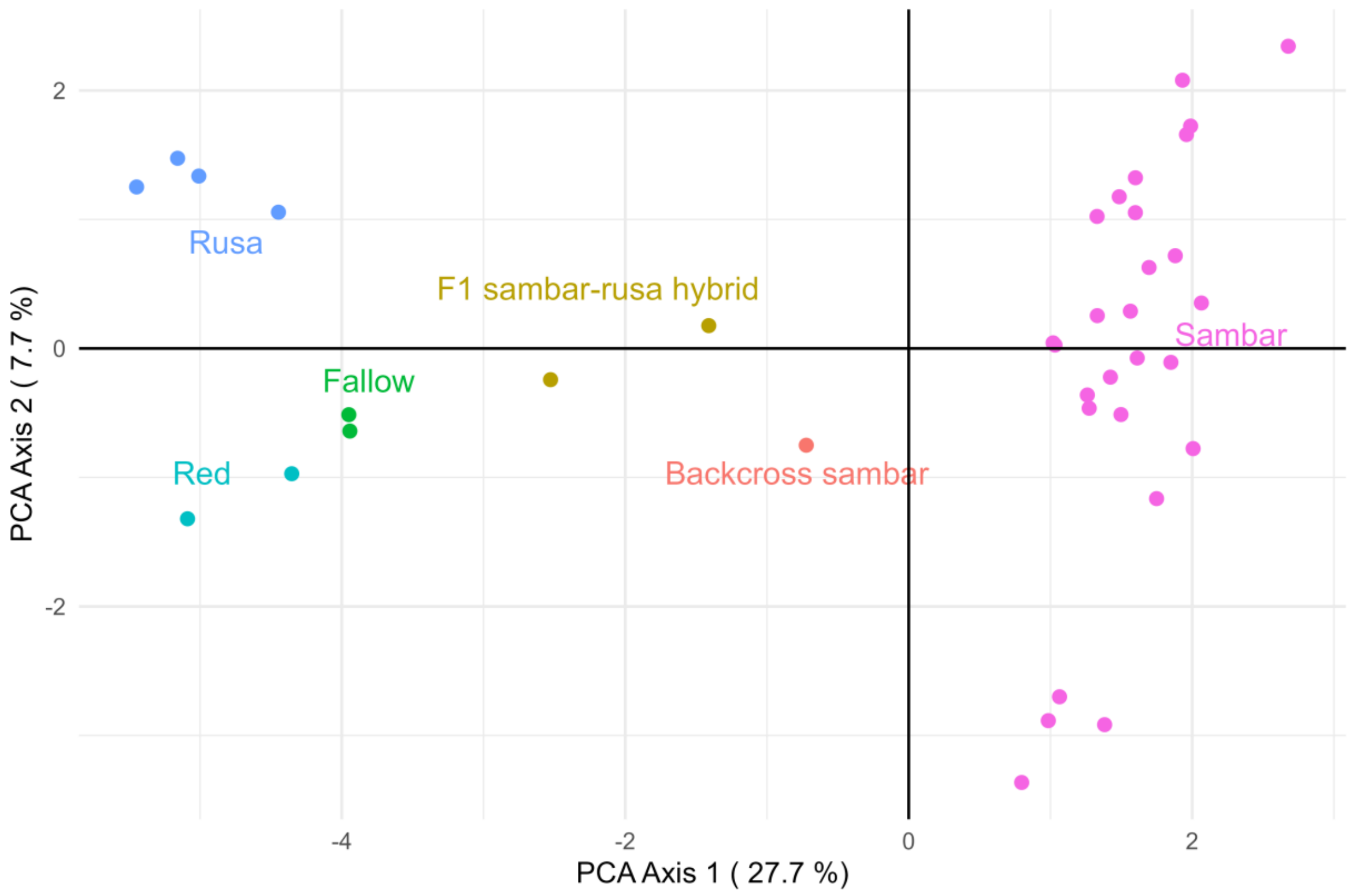

Figure S1. Principal Component Analysis (PCA) for 38 samples of four species used in panel development, genotyped at 220 SNPs with 15.2% missing data. Sambar-rusa hybrids are labelled according to NewHybrids analysis by Hill, Murphy, Li-Williams, et al. (2023).

Figure S2 – Pilot study

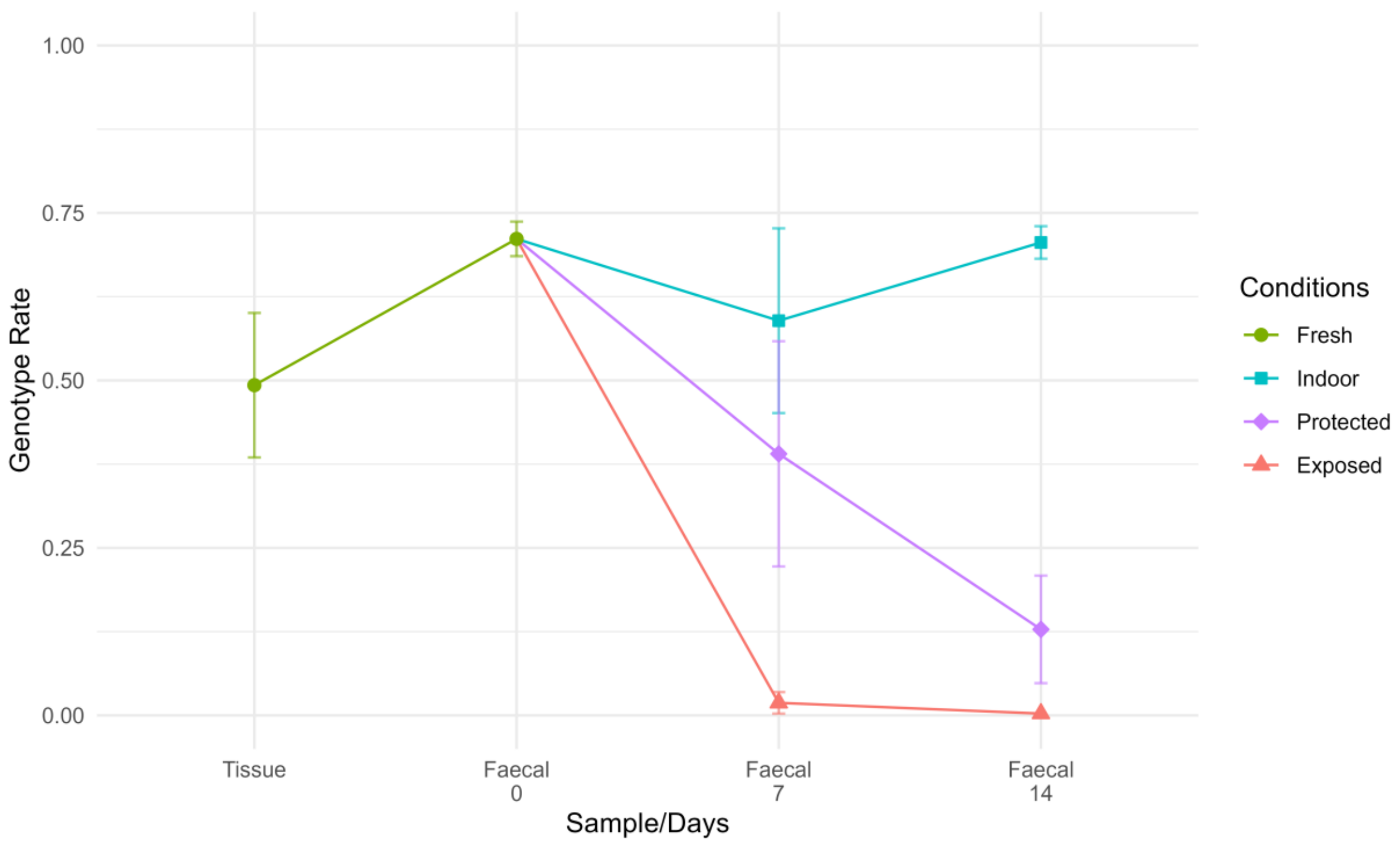

Figure S2. Mean genotype rate for fresh tissue samples and for faecal samples subjected to various conditions and amounts of time in the pilot degradation study, which included three individuals. Sequencing depth was lower in the pilot study. Note that *n*=1 for day 14 exposed faecal samples due to suspected coprophagy but missing values were inferred as close to zero based on day 7.

Figure S3 – Read depth and concentration over time

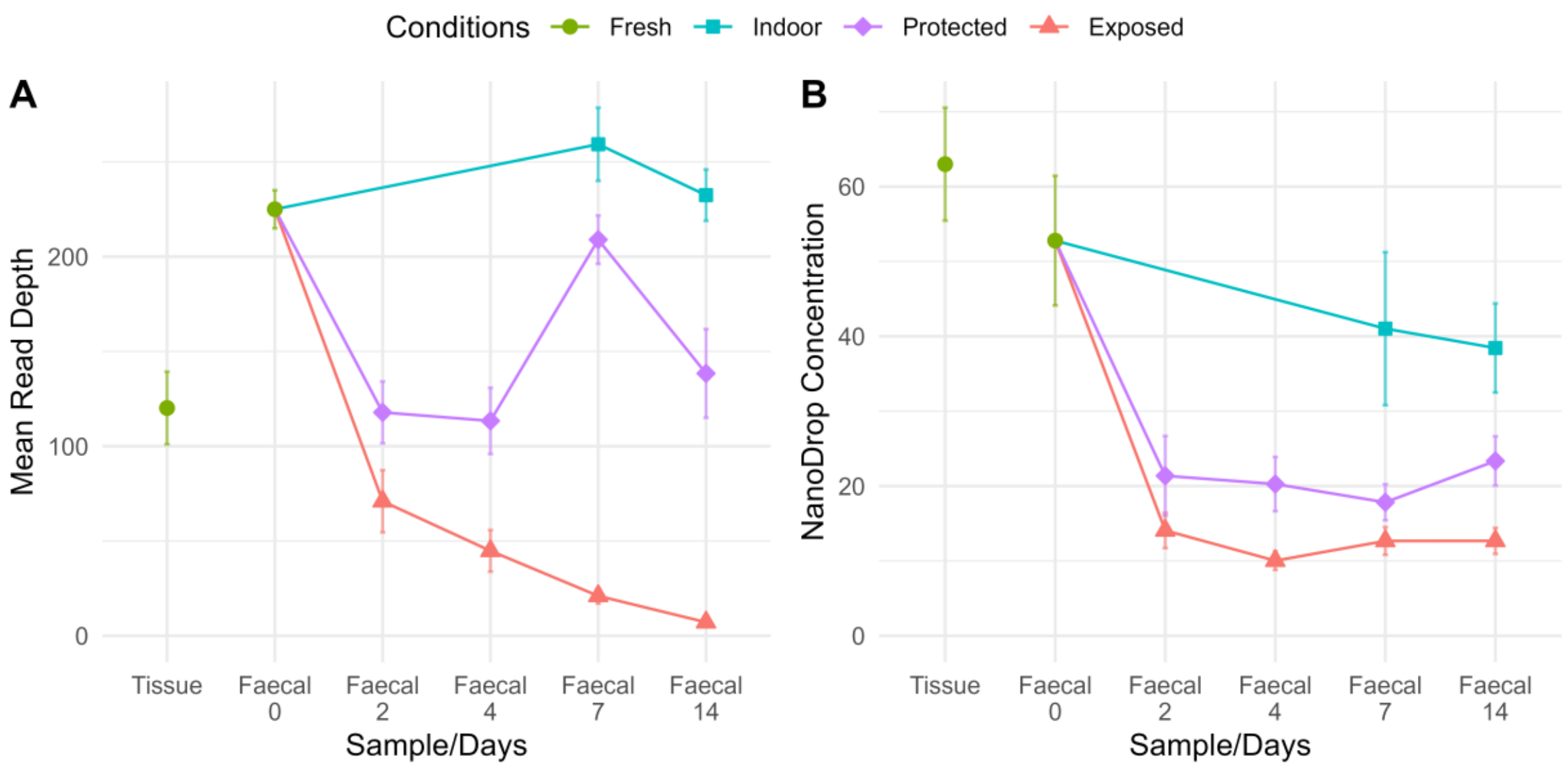

Figure S3. (A) Mean read depth of genotyped loci for fresh tissue samples and for faecal samples subjected to various conditions and amounts of time. (B) Mean NanoDrop concentration (ng/μL) prior to normalisation. Error bars represent standard error of the mean in both panels.

Figure S4 – Relationship between NanoDrop concentration and genotype rate

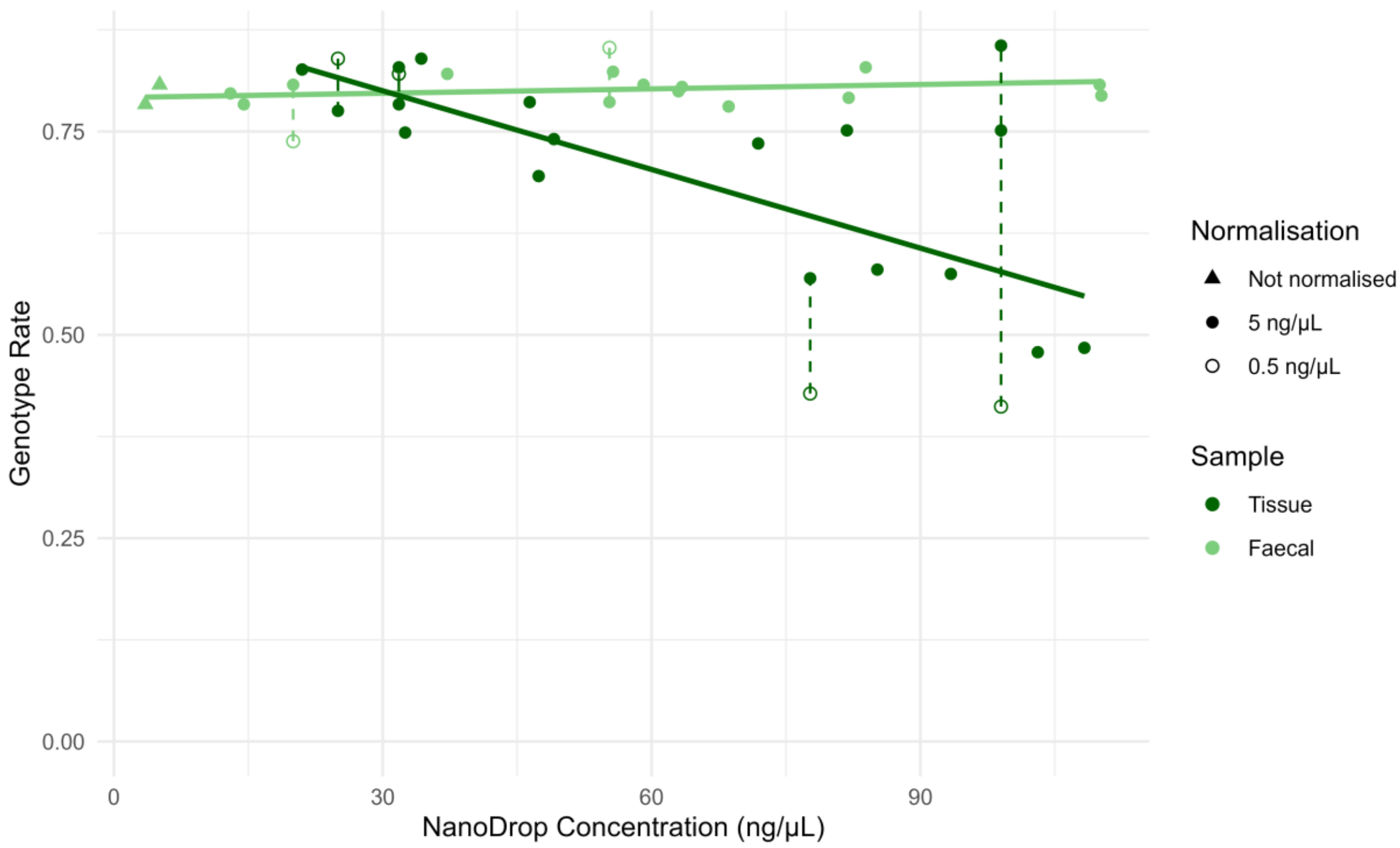

Figure S4. Relationship between NanoDrop concentration and genotype rate for tissue and fresh faecal samples. Dilution replicates (*n* = 6) which were normalised to 0.5 ng/uL are connected to their corresponding sample(s) with a dashed line. Tissue samples were normalised to 10 ng/µL but with PCR reactions adjusted accordingly so are considered equivalent to 5 ng/µL.

Figure S5 – Appearance of faecal pellets

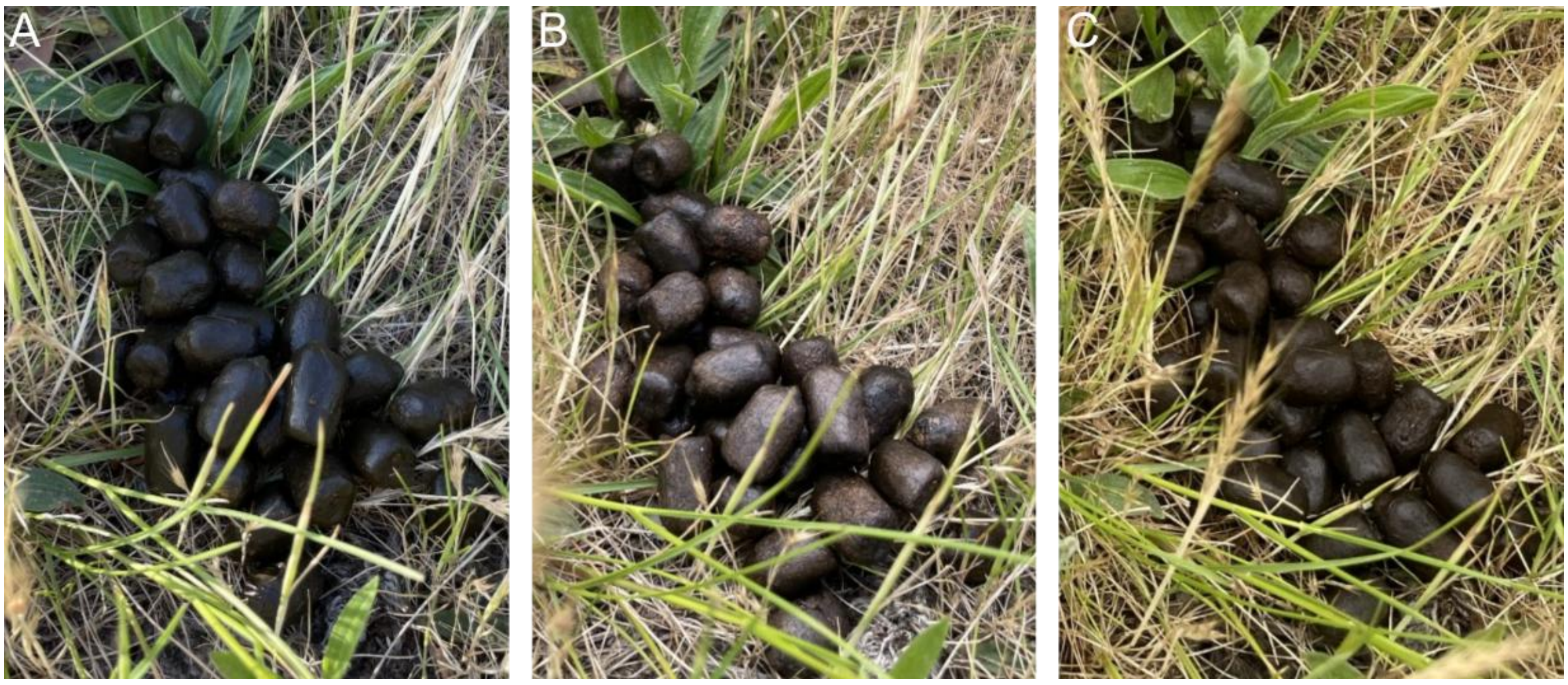

Figure S5. Appearance of the same group of pellets on (A) day 0, (B) day 1 and (C) day 4 of the degradation study.

Figure S6 – Read depth and genotyping relationship

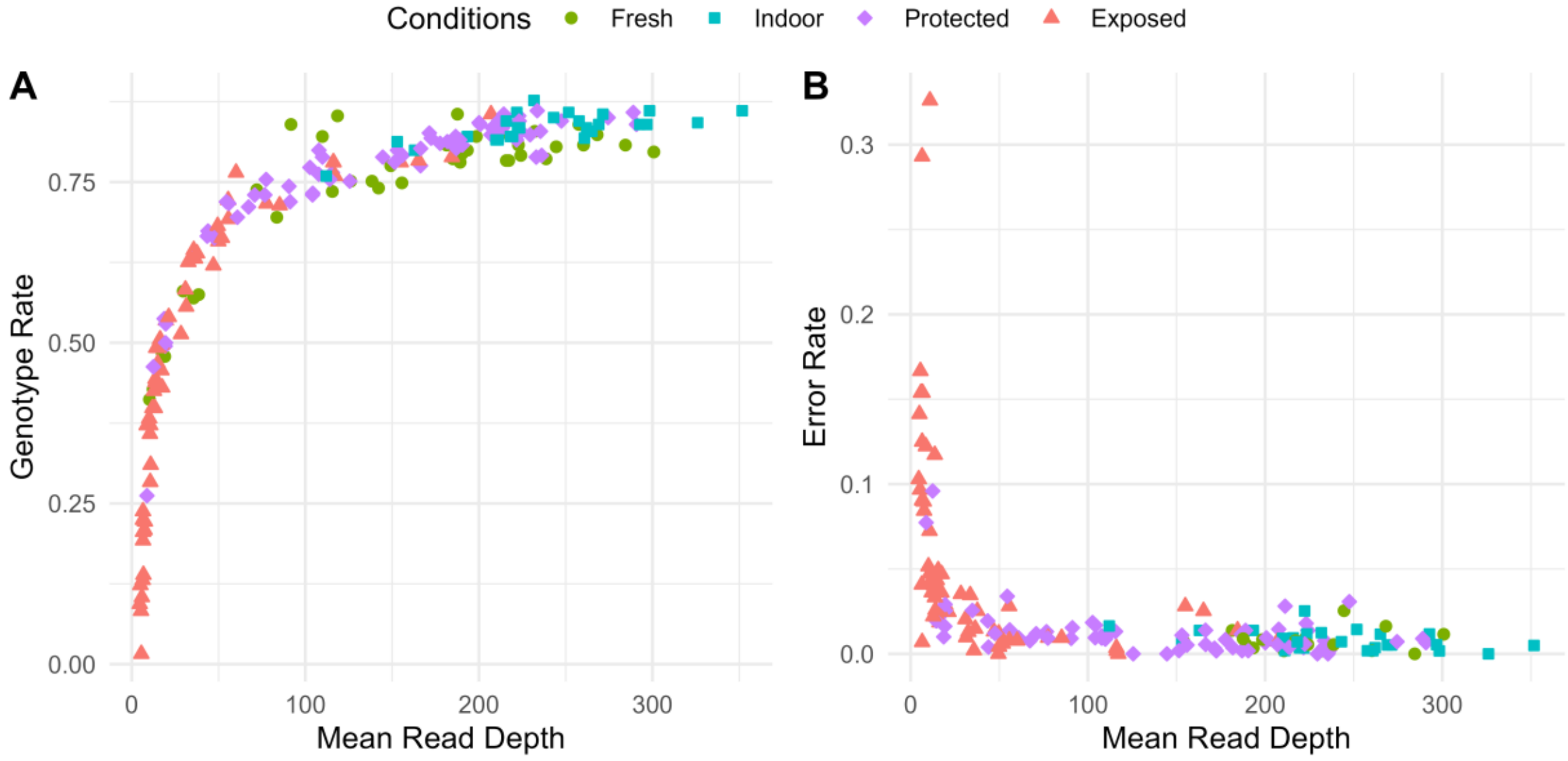

Figure S6. Relationship between sample mean read depth and (A) genotype rate and (B) error rate.

Figure S7 – Structure for different K

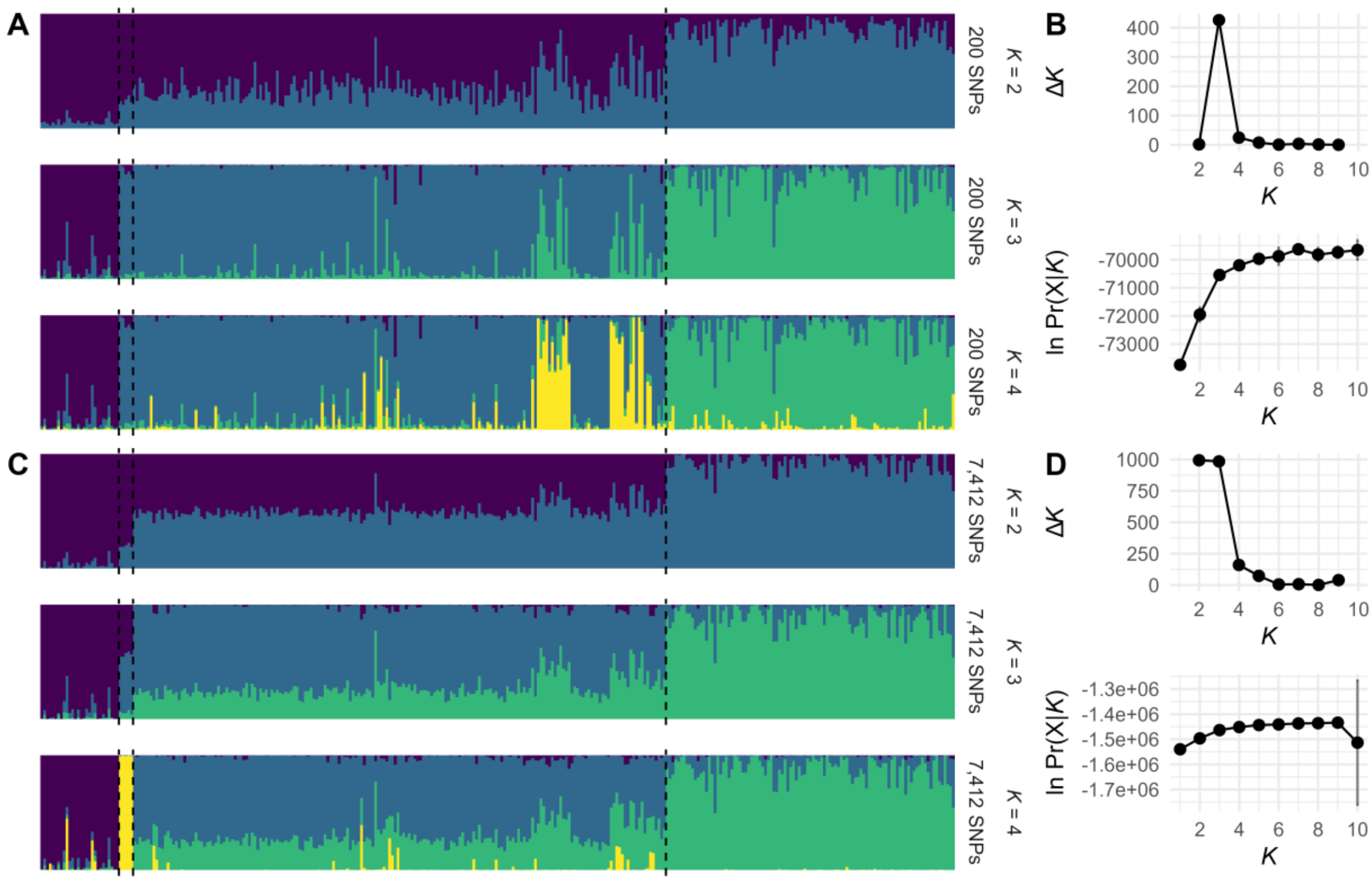

Figure S7. (A) *Structure* plot for *K*=2, *K*=3, and *K*=4 for the 200 SNP dataset. From left to right, the four groups indicated by dotted lines are Northern NSW, Werakata NP, Gippsland and Melbourne. (B) Evanno et al. (2005) method and ln Pr(X|*K*) method (Pritchard et al., 2000) plots for the 200 SNP dataset. (C) *Structure* plot for *K*=2, *K*=3, and *K*=4 for the 7,412 SNP dataset. (D) Plots as in (B) for the 7,412 SNP dataset.

References

Evanno, G., Regnaut, S., & Goudet, J. (2005). Detecting the number of clusters of individuals using the software STRUCTURE: a simulation study. *Molecular Ecology*, *14*(8), 2611-2620. https://doi.org/10.1111/j.1365-294X.2005.02553.x

Gruber, B., Unmack, P. J., Berry, O. F., & Georges, A. (2018). dartr: An r package to facilitate analysis of SNP data generated from reduced representation genome sequencing. *Molecular Ecology Resources*, *18*(3), 691-699. https://doi.org/10.1111/1755-0998.12745

Gurgul, A., Radko, A., & Słota, E. (2009). Characteristics of X- and Y-chromosome specific regions of the amelogenin gene and a PCR-based method for sex identification in red deer (*Cervus elaphus*). *Molecular Biology Reports*, *37*(6), 2915-2918. https://doi.org/10.1007/s11033-009-9852-4

Hill, E., Murphy, N., Li-Williams, S., Davies, C., Forsyth, D., Comte, S., Rollins, L., Hogan, F., Wedrowicz, F., Crittle, T., Thomas, E., Woodford, L., & Pacioni, C. (2023). Hybridisation rates, population structure, and dispersal of sambar deer (*Cervus unicolor*) and rusa deer (*Cervus timorensis*) in south-eastern Australia. *Wildlife Research*. https://doi.org/10.1071/wr22129

Hill, E., Pacioni, C., White, L. C., D'Ombrain, T. H., & Murphy, N. P. (in prep.). Population structure of sambar deer (*Cervus unicolor*) is influenced by close relatives in the peri-urban region of Melbourne, Australia. *Unpublished manuscript*.

Kim, B. J., Lee, H., & Lee, S.-d. (2009). Species- and sex-specific multiple PCR amplifications of partial cytochrome *b* gene and *Zfx/Zfy* introns from invasive and non-invasive samples of Korean ungulates. *Genes & Genomics*, *31*(5), 369-375. https://doi.org/10.1007/bf03191255

Li-Williams, S., Stuart, K. C., Comte, S., Forsyth, D. M., Dawson, M., Sherwin, W. B., Rollins, L. A., & Nugent, G. (2023). Genetic analysis reveals spatial structure in an expanding introduced rusa deer population. *Wildlife Research*, *50*(9), 757-769. https://doi.org/10.1071/wr22128

Mijangos, J. L., Gruber, B., Berry, O., Pacioni, C., & Georges, A. (2022). dartR v2: An accessible genetic analysis platform for conservation, ecology and agriculture. *Methods in Ecology and Evolution*, *13*(10), 2150-2158. https://doi.org/10.1111/2041-210x.13918

Pritchard, J. K., Stephens, M., & Donnelly, P. (2000). Inference of population structure using multilocus genotype data. *Genetics*, *155*(2), 945-959. https://doi.org/10.1093/genetics/155.2.945

Rollins, L. A., Lees, D., Woolnough, A. P., West, A. J., Perry, M., Forsyth, D. M., & Pople, T. (2023). Origins and population genetics of sambar deer (*Cervus unicolor*) introduced to Australia and New Zealand. *Wildlife Research*, *50*(9), 716-727. https://doi.org/10.1071/wr22120

Waits, L. P., Luikart, G., & Taberlet, P. (2001). Estimating the probability of identity among genotypes in natural populations: cautions and guidelines. *Molecular Ecology*, *10*(1), 249-256. https://doi.org/10.1046/j.1365-294x.2001.01185.x

White, L. C., Hill, E., Murphy, N., Ramsey, D. S. L., Woodford, L., Schneider, T., Bennett, A., McMaster, D., Dahal, K., Pacioni, C., & Prowse, T. (2025). Quantifying deer control efficiency using non-invasive genetic sampling and spatial capture–recapture. *Wildlife Research*, *52*(10). https://doi.org/10.1071/wr24214

Yamauchi, K., Hamasaki, S.-I., Miyazaki, K., Kikusui, T., Takeuchi, Y., & Mori, Y. (2000). Sex determination based on fecal DNA analysis of the amelogenin gene in sika deer (*Cervus nippon*). *The Journal of Veterinary Medical Science*, *62*(6), 669–671. https://doi.org/10.1292/jvms.62.669
